# Synthesis and Antiproliferative Activity of Novel A-Ring Cleaved Glycyrrhetinic Acid Derivatives

**DOI:** 10.3390/molecules24162938

**Published:** 2019-08-14

**Authors:** Daniela P.S. Alho, Jorge A.R. Salvador, Marta Cascante, Silvia Marin

**Affiliations:** 1 Laboratory of Pharmaceutical Chemistry, Faculty of Pharmacy, University of Coimbra, 3000-548 Coimbra, Portugal; 2 Centre for Neuroscience and Cell Biology, 3000-504 Coimbra, Portugal; 3 Department of Biochemistry and Molecular Biomedicine, Faculty of Biology, University of Barcelona, Diagonal 643, 08028 Barcelona, Spain; 4 Centro de Investigación Biomédica en Red de Enfermedades Hepáticas y Digestivas (CIBEREHD), Instituto de Salud Carlos III (ISCIII), 28029 Madrid, Spain

**Keywords:** pentacyclic triterpenoids, glycyrrhetinic acid, A-ring cleaved derivatives, antiproliferative activity, cell cycle arrest, apoptosis

## Abstract

A series of new glycyrrhetinic acid derivatives was synthesized via the opening of its ring A along with the coupling of an amino acid. The antiproliferative activity of the derivatives was evaluated against a panel of nine human cancer cell lines. Compound **17** was the most active compound, with an IC_50_ of 6.1 µM on Jurkat cells, which is 17-fold more potent than that of glycyrrhetinic acid, and was up to 10 times more selective toward that cancer cell line. Further biological investigation in Jurkat cells showed that the antiproliferative activity of compound **17** was due to cell cycle arrest at the S phase and induction of apoptosis.

## 1. Introduction

Cancer is a leading cause of death worldwide. Its global incidence continues to rise due to the aging and growth of the world population and the increasing adoption of lifestyle choices associated with cancer in developed countries [1]. Plants have been a major source of highly effective conventional drugs for cancer treatment and nowadays they play an important role as a source of leads for the development of potential new agents [2,3]. The plant-derived triterpenoids are proving to be interesting leading compounds as reported in a large number of scientific papers emerging in this field [4,5,6,7,8,9,10,11,12,13,14,15].

Glycyrrhetinic acid (GA) **1** is the hydrolyzed metabolite of glycyrrhizin, a major pentacyclic triterpenoid saponin obtained from the roots of licorice (*Glycyrrhiza* species) in high yields up to 24% [16,17]. This compound has been shown to inhibit tumor initiation [18,19,20,21,22] and proliferation in several cancer cell lines; its antiproliferative activity is mediated by cell cycle arrest [23,24] and induction of apoptosis [21,25,26,27,28]. The antitumor effects of GA **1** were also observed in animal models [20,29,30]. Nevertheless, it lacks potency and selectivity as an antitumor agent. Many derivatizations have been performed in order to enhance the potency of GA **1** [31,32,33,34,35,36]. However, the cleavage of its ring A is still poorly explored [37]. On the other hand, is well known that the conjugation of an amino acid moiety to pentacyclic triterpenoids improves their cytotoxicity and their selectivity towards tumor cells [38,39,40]. These findings prompted us to synthesize new GA **1** derivatives via the opening of its ring A along with the coupling with an amino acid. The novel semisynthetic derivatives were tested for their antiproliferative activity against a panel of nine human cancer cell lines. Further biological assays were conducted for the most potent compound **17** in the cancer cell line that yielded the best results (Jurkat cells), to investigate its preliminary mechanism of action. The study of selectivity was performed on human fibroblasts (BJ).

## 2. Results and Discussion

### 2.1. Chemistry

**Scheme 1:**
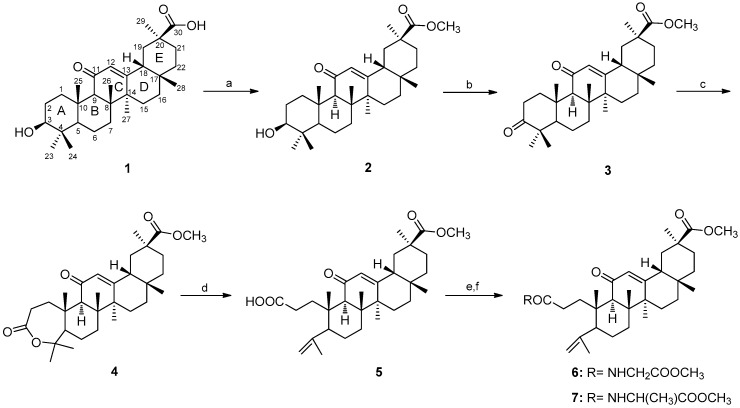
Reagents and conditions: (a) CH_3_I, K_2_CO_3_, DMF, r.t.; (b) Jones reagent, acetone, 0 °C; (c) *m*-CPBA, CH_2_Cl_2_, r.t.; (d) *p*-TSA, CH_2_Cl_2_, r.t.; (e) Deoxo-Fluor®, CH_2_Cl_2_, r.t.; (f) glycine methyl ester hydrochloride or L-alanine methyl ester hydrochloride, Et_3_N, CH_2_Cl_2_, r.t.

**Scheme 2:**
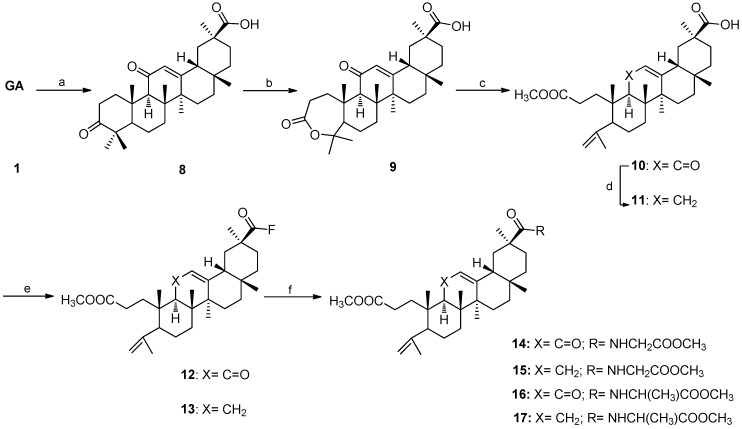
Reagents and conditions: (a) Jones reagent, acetone, 0 °C; (b) *m*-CPBA,CH_2_Cl_2_, r.t.; (c) MeOH, p-TSA, CH_2_Cl_2_, r.t.; (d) zinc dust, conc. HCl, dioxane, r.t.; (e) Deoxo-Fluor®, CH_2_Cl_2_, r.t.; (f) glycine methyl ester hydrochloride or L-alanine methyl ester hydrochloride, Et_3_N, CH_2_Cl_2_, r.t.

**Scheme 3:**
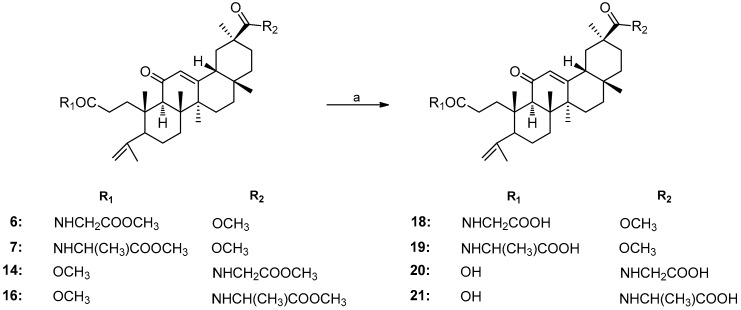
Reagents and conditions: (a) KOH 4N, THF/MeOH, r.t.

The synthesis of the glycyrrhetinic acid **1** derivatives is outlined in Scheme 1, Scheme 2 and Scheme 3. Full structural elucidation of the new glycyrrhetinic acid derivatives was achieved using nuclear magnetic resonance (NMR), mass spectrometry (MS) and elemental analysis. The analytical data obtained for the known compounds **1**–**5** and **8**–**10** were in agreement with those reported in the literature [39,41,42,43].

The synthesis of compounds **2**–**7** is summarized in Scheme 1. Methyl ester **2** was obtained from the reaction of compound **1**, the starting material, with methyl iodide in the presence of potassium carbonate [39]. The 3β-hydroxyl group of compound **2** was then oxidized using the Jones reagent [41] to give the 3-keto derivative **3**. The reaction of this derivative with *m*-chloroperbenzoic acid (*m*-CPBA) provided lactone **4**. The lactone ring of **4** was opened by treatment with *p*-toluenesulfonic acid (*p*-TSA) in dichloromethane [42]. Reaction of compound **5** with bis(2-methoxyethyl)aminosulfur trifluoride (Deoxo-Fluor^®^) [44] provided the acyl fluoride intermediate which was reacted either with glycine methyl ester hydrochloride or with L-alanine methyl ester hydrochloride to afford compounds **6** and **7**, in yields of 69% and 61%, respectively. We found that the acyl fluoride, in this position of the structure, decomposes on standing. For that reason, the crude compound was employed without further purification, and immediately, in the subsequent reactions. The preparation of compounds **6** and **7** was confirmed by the presence of the proton signals of the amino acid side chains. On the ^1^H NMR spectrum of compound **6**, the δ signals of the glycine methyl ester side chain were observed around 6.1 ppm (NH), 4.0 ppm (NCH_2_) and 3.7 ppm (CH_3_). Compound **7**, with an alanine methyl ester side chain, had δ signals around 6.1 ppm (NH), 4.6 ppm (NCH) and 3.7 ppm (CH_3_).

Compounds **8–17** were synthesized as depicted in Scheme 2. Compound **1** was oxidized using the Jones reagent [41] to afford compound **8**, which was reacted with *m*-CPBA to give the derivative **9**. The lactone ring of **9** was cleaved by treatment with *p*-TSA in methanol and dichloromethane [42] to provide compound **10**. The derivative **11** and the three pairs of compounds synthesized in the following steps were prepared to explore the influence of the keto group in position C-11 on the antiproliferative activity. The removal of the keto group was performed by a Clemmensen reduction [45] with zinc dust and concentrated HCl in dioxane at room temperature to afford **11** (75%). The reduction was confirmed on the ^13^C NMR spectrum, by the absence of the δ signal around 200 ppm, which corresponds to the carbonyl group in ring C. Acyl fluorides **12** and **13** were obtained from the reaction of compounds **10** and **11** with Deoxo-Fluor, in yields of 75% and 61%, respectively. The synthesis of acyl fluorides was detected on the ^13^C NMR spectra. The carbon C30 appeared as a doublet with a δ signal around 166 ppm and a coupling constant of 375 Hz, in both compounds **12** and **13**. These derivatives were reacted either with glycine methyl ester hydrochloride or with L-alanine methyl ester hydrochloride to afford compounds **14**–**17**, in yields ranging from 43% to 83%. The glycine methyl ester side chain of compounds **14** and **15** was detected on the ^1^H NMR spectra. Its proton signals were observed around 6.2 ppm (NH), 4.0 ppm (NCH_2_) and 3.8 ppm (CH_3_). Compounds **16** and **17**, with an alanine methyl ester side chain, had δ signals around 6.2 ppm (NH), 4.6 ppm (NCH) and 3.8 ppm (CH_3_).

Deprotection of the carboxyl group of the amino acid chain was performed in compounds **6**, **7**, **14** and **16**, by alkaline hydrolysis (Scheme 3). This reaction also caused deprotection of the other carboxyl group on compounds **14** and **16**. Compounds **18**–**21** were obtained in yields ranging from 94% to 98%. Deprotection of the carboxyl group of the amino acid chains was confirmed by the absence of δ signals around 3.7–3.8 ppm, on the ^1^H NMR spectra of compounds **18**–**21**. The loss the other methyl group of compounds **14** and **16** was detected by the absence of the δ signal around 3.6 ppm, on the ^1^H NMR spectra of compounds **20** and **21**.

### 2.2. Biology

#### 2.2.1. Antiproliferative Activity

Several cancer cell lines were used to evaluate the potential cytotoxicity of the synthesized compounds against human cancers. This evaluation was based on the determination of the concentration that inhibits cell proliferation at 50% (IC_50_), using 3-(4,5-dimethylthiazol-2-yl)-3,5-diphenyltetrazolium bromide (MTT) or 2,3-bis(2-methoxy-4-nitro- 5-sulphophenyl)-2*H*-tetrazolium-5-carboxanilide (XTT) assays, after 72 h of treatment with the compounds.

Compounds **1**, **5**–**7** and **10**–**21** were screened for their antiproliferative activity on A549 (lung adenocarcinoma) and HT-29 (colon adenocarcinoma) cell lines (Table 1). Compounds **2**–**4**, **8** and **9** were not evaluated because they have already been tested with no improvements in potency and/or selectivity [37,39,46,47]. Intermediates **5** and **10**, afforded by the cleavage of the ring A, were more potent than the parental compound GA **1**. Removal of the keto group from ring C, that provided compound **11**, resulted in an increment of cytotoxicity. Acyl fluorides **12** and **13** were less potent compared to their substrates. Analysis of the IC_50_ values of the derivatives **6, 7** and **14**–**17** showed that the conjugation of an amino acid methyl ester provided more potent compounds. Deprotection of the carboxyl groups resulted in a loss of cytotoxicity. Compounds **18**–**21**, afforded by the alkaline hydrolysis, were further tested in Jurkat (acute T-cell leukemia) and MOLT-4 (acute lymphoblastic leukemia) cell lines (Table 2). The results of these assays confirmed that the deprotection provided less active compounds. Derivatives **6, 7** and **14**–**17** and the parental compound GA **1** were also screened for their antiproliferative activity in seven additional human cancer cell lines: Jurkat, MOLT-4, MIAPaca 2 (pancreas adenocarcinoma), MCF7 (breast adenocarcinoma), HeLa (cervix adenocarcinoma), A375 (melanoma) and HepG2 (hepatocellular carcinoma). Comparing IC_50_ values of compounds **6** and **7** with those obtained for compounds **14** and **16**, no significant differences were found regarding the position in which the amino acid methyl ester was introduced. Derivatives **15** and **17** were respectively more potent than compounds **14** and **16** in all tested cell lines, which confirmed that the removal of the keto group from ring C enhanced the cytotoxicity. Compounds **7**, **16** and **17,** which have an alanine methyl ester chain, were more active than compounds **6**, **14** and **15,** with a glycine methyl ester chain, respectively. These results suggest that the type of amino acid moiety introduced influences the antiproliferative activity. Within the newly synthesized derivatives, compound **17**, with a reduced ring C and with an alanine methyl ester chain, was the most potent derivative. This compound was 5 to 17-fold more active than GA **1**, depending on the cancer cell line.

The selectivity towards cancer cells was studied for GA **1** and compound **17** by incubating them with a human nontumoral cell line (BJ) (Table 2). GA **1** and compound **17** showed IC_50_ values that were 1.6 and more than 16.4 times lower on Jurkat cells than on the nontumoral BJ cells, respectively. Therefore, the novel derivative **17** was up to 10 times more selective towards malignant cells than its parental compound **1**. This compound also showed a significant improvement in selectivity compared to the chemotherapy agent cisplatin. Considering also the Jurkat cell line, cisplatin presented an IC_50_ value that was 5.3 times lower than on BJ cells (Table 2); therefore, compound **17** was up to 3 times more selective than cisplatin towards Jurkat cells.

#### 2.2.2. Analysis of Cell Cycle Distribution and Apoptosis

**Figure 1:**
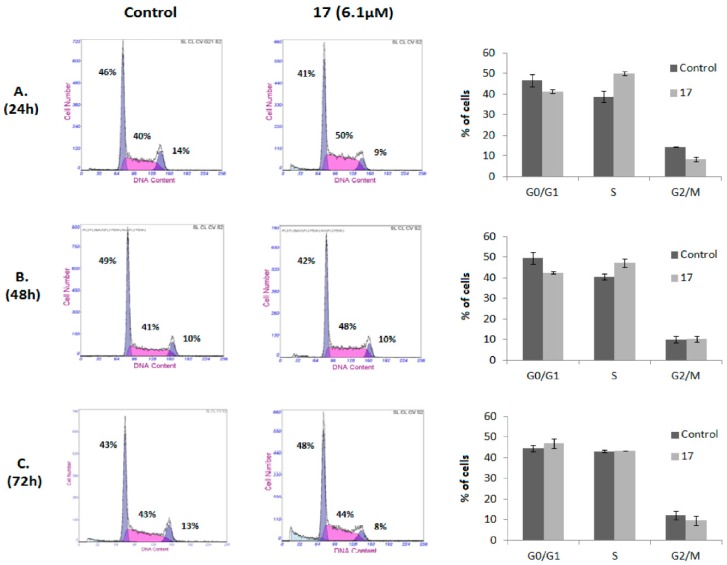
Effect of compound **17** on cell cycle distribution. Cell cycle analysis of Jurkat cells untreated (Control) or treated with 6.1 μM compound **17** for 24 h (**A**), 48 h (**B**), and 72 h (**C**). After treatment, cells were stained with PI and DNA content analyzed by flow cytometry. A representative histogram is shown for each incubation time and condition. Results are presented as means ± SD of three independent experiments.

The Jurkat cell line which was the most susceptible to these derivatives was selected to investigate the mechanism of action of compound **17**. To evaluate the effects on the cell cycle distribution, Jurkat cells were treated with compound **17**, at a concentration corresponding to its IC_50_ value at 72 h of treatment, for 24, 48 and 72 h and then analyzed by flow cytometry. The calculation of the fraction of cells in G0/G1, S and G2/M phases was performed using the fraction of live cells. Treatment for 24 h induced significant increase in the population at S phase with respect to untreated cells (Figure 1); after 48 h this effect has decreased and after 72 h it was no longer observed. DNA fragmentation was detected after 72 h of incubation based on the appearance of a sub-G0 peak. This sequence of events suggests that the cell cycle arrest at S phase may have led cells to undergo apoptosis. 

**Figure 2:**
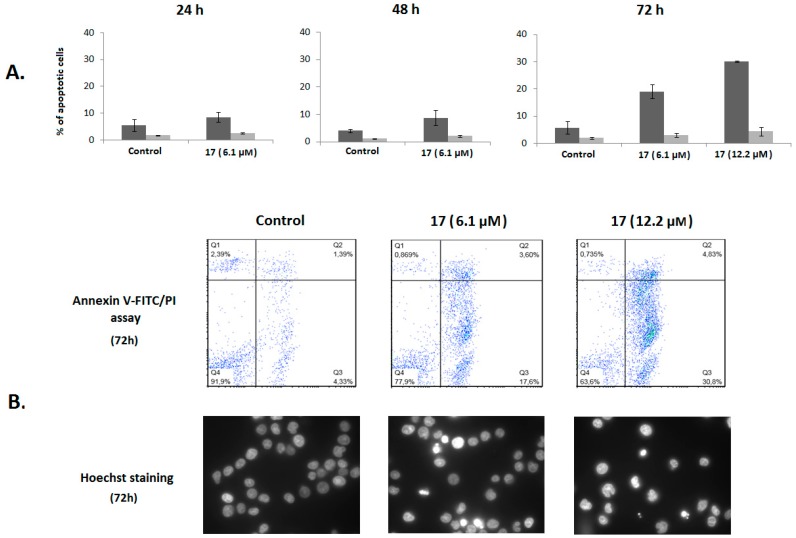
Induction of apoptosis by compound **17**. (**A**) Flow cytometry quantification of apoptosis in Jurkat cells untreated (Control) or treated with compound **17** at specified concentrations for 24, 48 and 72 h. After treatment, cells were stained with annexin V-FITC/PI and analyzed by flow cytometry. The percentage of early (dark gray bar) and late (light gray bar) apoptotic cells in each condition is represented as a bar diagram, calculated from dot plots. Results are presented as means ± SD of three independent experiments. (**B**) *Upper panel*: Representative dot plots of annexin V-FITC/PI assays of Jurkat cells untreated (Control) or treated with compound **17** at specified concentrations for 72 h; the right quadrants of each diagram (annexin^+^/PI^−^ and annexin^+^/PI^+^) represent apoptotic cells. *Lower panel*: Representative fluorescence microscopic images of Jurkat cells untreated (Control) or treated with compound **17** at specified concentrations for 72 h; Jurkat cells were stained with Hoechst 33342 before analysis by fluorescence microscopy.

Apoptosis assays were then performed to better elucidate the mechanism of cell death involved in the cytotoxic effect of compound **17**. The Annexin V-FITC/PI flow cytometric assay employs the property of fluorescein isothiocyanate (FITC) conjugated to Annexin V (Annexin V-FITC) to bind to phosphatidylserine (PS) and the property of propidium iodide (PI) to enter cells with damaged cell membranes and to bind to DNA. Early apoptosis is characterized by the loss of membrane asymmetry, with translocation of PS from the inner to the outer membrane, prior to the loss of membrane integrity. Therefore, this assay allows the discrimination of live cells (Annexin-V^−^/ PI^−^) from early apoptotic (Annexin-V^+^/PI^−^), late apoptotic (Annexin-V^+^/PI^+^) or necrotic cells (Annexin-V^+^/PI^+^). The experiments were conducted on Jurkat cells treated with compound **17** at a concentration corresponding to its IC_50_ value at 72 h of treatment (6.1 μM) for 24 and 48 h, and at concentrations of 6.1 μM and 12.2 μM for 72 h. Exposure to this compound for 24 and 48 h did not change significantly the apoptotic (Figure 2A) and necrotic (data not shown) populations. Treatment for 72 h with compound **17** at concentrations of 6.1 μM and 12.2 μM increased the early apoptotic population by 19% and 30%, respectively. No significant changes were observed in the late apoptotic population. These results were in good agreement with those obtained in the cell cycle experiments.

The induction of apoptosis was further confirmed by the observation of its characteristic morphological changes. Hoechst 33342 staining showed volume reduction, chromatin condensation and apoptotic bodies in Jurkat cells treated in the same conditions for 72 h (Figure 2B). In contrast, untreated cells presented a normal morphological profile.

Taken together, the results described above suggest that compound **17** inhibits cell growth through cell cycle arrest at the S phase and induction of apoptosis. Its mechanism of action needs to be studied further. 

## 3. Materials and Methods 

### 3.1. Chemistry

Glycyrrhetinic acid and all reagents were purchased from Sigma-Aldrich Co. (Saint Louis, MO, USA) The solvents used in the reactions were obtained from Merck Co. (Kenilworth, NJ, USA) and were purified and dried according to the literature procedures. The solvents used in workups were purchased from VWR Portugal. Thin layer chromatography (TLC) analysis was performed in Kieselgel 60HF254/Kieselgel 60G. Purification of compounds by flash column chromatography (FCC) was carried out using Kiesegel 60 (230–400 mesh, Merck). Melting points were determined using a BUCHI melting point B-540 apparatus and were uncorrected. ^1^H and ^13^C NMR spectra were recorded on a Bruker Avance-400 Digital NMR spectrometer, in CDCl_3,_ with Me_4_Si as the internal standard (see Supplementary Materials). Chemical shifts values (*δ*) are given in parts per million (ppm) and coupling constants (*J*) are presented in hertz (Hz). Mass spectra were obtained using a Quadrupole/Ion Trap Mass Spectrometer (QIT-MS) (LCQ Advantage MAX, THERMO FINNINGAN). Elemental analysis was performed in an Analyzer Elemental Carlo Erba 1108 by chromatographic combustion.

*Methyl 3β-hydroxy-11-oxo-olean-12-en-30-oate***(2)**: Compound **2** was prepared according to the literature [39], from **1** to give a colorless solid (90%). m.p.: 254–256 °C.

*Methyl 3,11-dioxo-olean-12-en-30-oate***(3)**: Preparation of **3** was performed according to a previously described method [41], from **2** providing a white solid (94%). m.p.: 248–250 °C.

*Methyl 3,11-dioxo-4-oxa-A-homo-olean-12-en-30-oate***(4)**: Compound **4** was prepared according to the literature [42], from **3** to give a white solid (77%). m.p.: 168–170 °C.

*3,4-seco-30-methyloxicarbonyl-11-oxo-olean-4(23),12-dien-3-oic acid***(5)**: Preparation of **5** was performed according to a previously described method [42], from **4** providing a white solid (72%). m.p.: 90–92 °C.

*Methyl 3,4-seco-3-N-methylglycinamido-11-oxo-olean-4(23),12-dien-30-oate***(6)**: To a solution of compound **5** (300 mg, 0.60 mmol) in dichloromethane (8 mL), Deoxo-Fluor (50% in THF, 0.52 mL, 1.20 mmol) was added and the reaction mixture was stirred, at room temperature, for 2.5 h, after which additional Deoxo-Fluor (50% in THF, 0.26 mL, 0.60 mmol) was added. After 2 h, the reaction was completed. The reaction mixture was quenched by addition of water (2 mL). The organic layer was diluted with chloroform (40 mL), washed with water (2 × 30 mL), dried over Na_2_SO_4_, filtered and evaporated to the dryness (280 mg, 93%). The residue was dissolved in dichloromethane (6 mL) and glycine methyl ester hydrochloride (105 mg, 0.84 mmol) and triethylamine (0.15 mL, 1.12 mmol) were added. After 1 h under magnetic stirring at room temperature, the reaction was completed. The reaction mixture was evaporated to dryness and ethyl acetate (40 mL) and water (30 mL) were added to the residue. The aqueous phase was further extracted with ethyl acetate (2 × 40 mL). The combined organic phase was washed with 5% aqueous HCl (2 × 30 mL), 10% aqueo+us NaHCO_3_ (2 × 30 mL), water (30 mL) and brine (30 mL), dried over Na_2_SO_4_, filtered and the solvent was removed under reduced pressure to afford a white solid. The solid was purified by flash column chromatography (FCC) with petroleum ether/ethyl acetate (2:1) to afford compound **6** as a white solid (69%). m.p.: 199–201 °C. ^1^H NMR (400 MHz, CDCl_3_): δ 6.14 (1H, m, NH), 5.69 (1H, s, H-12), 4.90 (1H, br s, H-23), 4.73 (1H, br s, H-23), 3.93-4.05 (2H, m, NCH_2_), 3.74 (3H, s, COOCH_3_), 3.69 (3H, s, COOCH_3_), 1.76 (3H, s), 1.38 (3H, s), 1.16 (3H, s), 1.15 (6H, s), 0.81 (3H, s). ^13^C NMR (100 MHz, CDCl_3_): δ 200.3 (C11), 177.1, 173.7, 170.6, 170.3, 146.7, 128.6 (C12), 114.5 (C23), 53.3, 52.4, 51.9, 51.3, 48.5, 45.2, 44.2, 43.8, 41.4, 41.3, 39.1, 37.9, 35.7, 32.0, 31.8, 31.5, 31.3, 28.7, 28.4, 26.7, 26.6, 24.0, 23.6, 23.5, 19.7, 18.8. ESI-MS m/z: 570.34 ([M + H] ^+^, 100%). Found C 70.78, H 9.39, N 2.41, calcd for C_34_H_51_NO_6_·0.25H_2_O: C 71.11, H 9.04, N 2.44%.

*Methyl 3,4-seco-3-N-methyalaninamido-11-oxo-olean-4(23),12-dien-30-oate***(7)**: The method followed followed that described for compound **6**. The resulting solid (274 mg, 0.55 mL; 91%) from the first step was dissolved in dichloromethane (6 mL) and alanine methyl ester hydrochloride (116 mg, 0.83 mmol) and triethylamine (0.15 mL, 1.10 mmol) were added. After 1 h, the reaction was completed. The workup was performed as described for compound **6**. The resulting solid was subjected to FCC with petroleum ether/ethyl acetate (2:1) to afford compound **7** as a white solid (61%). m.p.: 193–195 °C. ^1^H NMR (400 MHz, CDCl_3_): δ 6.09 (1H, m, NH), 5.70 (1H, s, H-12), 4.91 (1H, br s, H-23), 4.74 (1H, br s, H-23), 4.55 (1H, m, -NCH(CH_3_)-), 3.73 (3H, s, COOCH_3_), 3.69 (3H, s, COOCH_3_), 1.77 (3H, s), 1.38 (3H, s), 1.37 (3H, m, -NCH(CH_3_)-), 1.17 (3H, s), 1.15 (3H, s), 1.15 (3H,s), 0.82 (3H, s). ^13^C NMR (100 MHz, CDCl_3_): δ 200.3 (C11), 177.1, 173.8, 173.0, 170.2, 146.7, 128.6 (C12), 114.5 (C23), 53.3, 52.5, 51.9, 51.1, 48.5, 48.1, 45.2, 44.2, 43.9, 41.4, 39.1, 37.9, 35.8, 32.0, 31.9, 31.5, 31.3, 28.8, 28.4, 26.7, 26.6, 24.0, 23.7, 23.5, 19.7, 18.8, 18.5. ESI-MS m/z: 584.29 ([M + H] ^+^, 100%). Found C 71.40, H 9.31, N 2.32, calcd for C_35_H_53_NO_6_·0.25H_2_O: C 71.46, H 9.17, N 2.38 %.

*3,11-Dioxo-olean-12-en-30-oic acid***(8)**: Compound **8** was prepared according to the literature [41], from **1** to give a colorless solid (92%). m.p.: 308–310 °C.

*3,11-Dioxo-4-oxa-A-homo-olean-12-en-30-oic acid***(9)**: Compound **9** was prepared from **8**, using the same method as for the preparation of **4**, with the obtention of a white solid. (75%). m.p.: 268–270 °C.

*3,4-seco-3-methyloxicarbonyl-11-oxo-olean-4(23),12-dien-30-oic acid***(10)**: Preparation of **10** was performed according to a previously described method [42], from **9** providing a colorless solid (62%). m.p.: 130–132 °C.

*3,4-seco-3-methyloxicarbonyl-olean-4(23),12-dien-30-oic acid***(11)**: Preparation of **11** was done according to a previously described method [45]. Compound **10** (900 mg, 1.80 mmol) was dissolved in dioxane (25 mL) and Zn powder (941 mg, 14.40 mmol) was added. Concentrated HCl (37%, 3.6 mL, 43.20 mmol) was added dropwise for 30 min with stirring. After 4.5 h under magnetic stirring at room temperature, the reaction was completed. The reaction mixture was filtered and the solvent was removed under pressure. Diethyl ether (75 mL) and water (60 mL) were added to the residue. The aqueous phase was further extracted with diethyl ether (2 × 70 mL). The combined organic extract was then washed with 5% aqueous HCl (2 × 50 mL), 10% aqueous NaHCO_3_ (2 × 50 mL), water (50 mL) and brine (50 mL), dried over Na_2_SO_4_, filtered and evaporated to the dryness. The resulting solid was purified by (FCC) with petroleum ether/ ethyl acetate (1:1) to afford compound **11** as a white solid (75%). m.p.: 138–140 °C. ^1^H NMR (400 MHz, CDCl_3_): δ 5.32 (1H, m, H-12), 4.87 (1H, br, s, H-23), 4.67 (1H, br, H-23), 3.65 (3H, s, COOCH_3_), 1.75 (3H, s), 1.21 (3H, s), 1.16 (3H, s), 1.02 (3H, s), 0.94 (3H, s), 0.82 (3H, s). ^13^C NMR (100 MHz, CDCl_3_): δ 183.1 (C30), 174.8 (C3), 147.6, 144.3, 122.8 (C12), 113.7 (C23), 51.8, 50.6, 48.2, 44.2, 42.7, 42.2, 39.7, 39.3, 38.4, 38.0, 34.1, 32.2, 31.5, 31.2, 28.8, 28.6, 28.3, 27.1, 26.2, 26.0, 24.7, 23.8, 23.6, 19.7, 17.0. ESI-MS m/z: 118.11 (19%), 274.50 (32), 318.46 (75), 346.48 (11), 362.45 (15), 485.38 ([M + H] ^+^, 100%).

*Methyl 3,4-seco-30-fluorcarbonyl-11-oxo-olean-4(23),12-dien-3-oate***(12)**: To a solution of compound **10** (500 mg, 1.00 mmol) in dichloromethane (10 mL), Deoxo-Fluor (50% in THF, 0.87 mL, 2.00 mmol) was added. After 1.5 h under magnetic stirring at room temperature, the reaction was completed. The reaction mixture was quenched by addition of water (3 mL). The organic layer was diluted with chloroform (50 mL), washed with water (2 × 40 mL), dried over Na_2_SO_4_, filtered and evaporated to the dryness. The resulting solid was subjected to (FCC) [petroleum ether/ethyl acetate from (6:1) to (4:1)] to afford compound **12** as a white solid (75%). m.p.: 183–185 °C. ^1^H NMR (400 MHz, CDCl_3_): δ 5.71 (1H, s, H-12), 4.90 (1H, br s, H-23), 4.69 (1H, br s, H-23), 3.62 (3H, s, COOCH_3_), 1.75 (3H, s), 1.38 (3H, s), 1.30 (3H, s), 1.17 (3H, s), 1.16 (3H, s), 0.86 (3H, s). ^13^C NMR (100 MHz, CDCl_3_): δ 199.5 (C11), 174.5 (C3), 168.1, 166.3 (J = 374.6 Hz, C30), 146.7, 129.1 (C12), 114.4 (C23), 53.0, 51.7, 51.0, 48.1, 45.3, 44.4 (J = 42.0 Hz), 43.8, 40.8, 38.9, 37.6, 34.6, 32.1, 31.6, 30.9, 29.4, 28.5, 27.0, 26.6, 26.5, 23.9, 23.6, 23.5, 19.7, 18.8. ESI-MS m/z: 501.16 ([M + H] ^+^, 100%).

*Methyl 3,4-seco-30-fluorcarbonyl-olean-4(23),12-dien-3-oate***(13)**: The method followed that described for compound **12** but using compound **11** (600 mg, 1.24 mmol) and Deoxo-Fluor (50% in THF, 1.08 mL, 2.48 mmol) in dichloromethane (12 mL) for 2 h. The resulting solid was purified by FCC [petroleum ether /ethyl acetate from (10:1) to (4:1)] to afford compound **13** as a white solid (61%). m.p.: 174–176 °C. ^1^H NMR (400 MHz, CDCl_3_): δ 5.32 (1H, m, H-12), 4.87 (1H, br s, H-23), 4.67 (1H, br s, H-23), 3.65 (3H, s, COOCH_3_), 1.75 (3H, s), 1.27 (3H, s), 1.15 (3H, s), 1.02 (3H, s), 0.94 (3H, s), 0.83 (3H, s). ^13^C NMR (100 MHz, CDCl_3_): δ 174.7 (C3), 167.0 (J = 375.0 Hz, C30), 147.5, 143.6, 123.4 (C12), 113.7 (C23), 51.7, 50.6, 48.3, 44.6 (J = 41.2 Hz), 42.4, 42.2, 39.7, 39.3, 38.1, 38.0, 34.1, 32.2, 31.5, 31.1, 28.6, 28.1, 27.2, 27.0, 26.1, 26.0, 24.6, 23.8, 23.6, 19.7, 17.0. ESI-MS m/z: 439.51 (38%), 440.52 (14), 485.44 (27), 487.36 ([M + H] ^+^, 100%).

*Methyl 3,4-seco-30-N-methylglycinamido-11-oxo-olean-4(23),12-dien-3-oate***(14)**: To a solution of compound **12** (300 mg, 0.60 mmol) and glycine methyl ester hydrochloride (226 mg, 1.80 mmol) in dichloromethane (8 mL), triethylamine (0.33 mL, 2.40 mmol) was added. After 13 h under magnetic stirring at room temperature, the reaction was completed. The reaction mixture was evaporated to dryness and ethyl acetate (50 mL) and water (40 mL) were added to the residue. The aqueous phase was further extracted with ethyl acetate (2 × 50 mL). The resulting organic phase was washed with 5% aqueous HCl (2 × 30 mL), 10% aqueous NaHCO_3_ (2 × 30 mL), water (30 mL) and brine (30 mL), dried over Na_2_SO_4_, filtered and the solvent was removed under reduced pressure to afford a solid. The solid was subjected to (FCC) [petroleum ether /ethyl acetate from (2:1) to (1:1)] to afford compound **14** as a white solid (77%). m.p.:126–128 °C. ^1^H NMR (400 MHz, CDCl_3_): δ 6.17 (1H, m, NH), 5.74 (1H, s, H-12), 4.88 (1H, br s, H-23), 4.68 (1H, br s, H-23), 3.95-4.14 (2H, m, NCH_2_), 3.76 (3H, s, COOCH_3_), 3.61 (3H, s, COOCH_3_), 1.75 (3H, s), 1.39 (3H, s), 1.16 (6H, s), 1.15 (3H, s), 0.83 (3H, s). ^13^C NMR (100 MHz, CDCl_3_): δ 199.7 (C11), 176.3, 174.5, 170.7, 169.5, 146.7, 128.6 (C12), 114.3 (C23), 52.9, 52.5, 51.7, 51.0, 47.9, 45.2, 43.8 (2), 42.1, 41.3, 38.9, 37.4, 34.6, 32.0, 31.6 (2), 29.5, 29.4, 28.6, 26.7, 26.5, 23.9, 23.6, 23.4, 19.6, 18.8. ESI-MS m/z: 570.32 ([M + H] ^+^, 100%). Found C 70.83, H 9.27, N 2.49, calcd for C_34_H_51_NO_6_·0.25H_2_O: C 71.11, H 9.04, N 2.44%.

*Methyl 3,4-seco-30-N-methylglycinamido-olean-4(23),12-dien-3-oate***(15)**: The method followed that described for compound **14** but using compound **13** (290 mg, 0.60 mmol), glycine methyl ester hydrochloride (226 mg, 1.80 mmol) and triethylamine (0.33 mL, 2.40 mmol) in dichloromethane (8 mL). The workup was performed after 16 h. The resulting solid was purified by FCC with petroleum ether/ ethyl acetate (3:2) to afford compound **15** as a white solid (43%). m.p.: 190–192 °C. ^1^H NMR (400 MHz, CDCl_3_): δ 6.18 (1H, m, NH), 5.34 (1H, m, H-12), 4.87 (1H, br s, H-23), 4.67 (1H, br s, H-23), 3.97-4.17 (2H, m, NCH_2_), 3.77 (3H, s, COOCH_3_), 3.65 (3H, s, COOCH_3_), 1.75 (3H, s), 1.17 (3H, s), 1.13 (3H, s), 1.01 (3H, s), 0.93 (3H, s), 0.79 (3H, s). ^13^C NMR (100 MHz, CDCl_3_): δ 177.0, 174.7, 171.0, 147.6, 144.4, 122.8 (C12), 113.7 (C23), 52.5, 51.7, 50.6, 48.1, 44.1, 43.6, 42.2, 41.4, 39.7, 39.3, 38.0 (2),34.2, 32.2, 31.7, 31.5, 29.8, 28.6, 28.3, 27.0, 26.2, 25.9, 24.6, 23.9, 23.6, 19.7, 17.0. ESI-MS m/z: 274.37 (25%), 318.32 (27), 556.24 ([M + H] ^+^, 100%). Found C 73.18, H 9.83, N 2.55, calcd for C_34_H_53_NO_5_: C 73.47, H 9.61, N 2.52 %.

*Methyl 3,4-seco-30-N-methylalaninamido-11-oxo-olean-4(23),12-dien-3-oate***(16)**: To a solution of compound **12** (300 mg, 0.60 mmol) and alanine methyl ester hydrochloride (251 mg, 1.80 mmol) in dichloromethane (8 mL), triethylamine (0.33 mL, 2.40 mmol) was added and the reaction mixture was stirred, at room temperature, for 8 h, after which additional dichloromethane (1 mL) and triethylamine (0.17 mL, 1.20 mmol) were added. After 16 h, the reaction was completed. The workup was performed as described for compound **14**. The solid was subjected to FCC with petroleum ether/ethyl acetate (1:2) to afford compound **16** as a white solid (83%). m.p.: 130–132 °C. ^1^H NMR (400 MHz, CDCl_3_): δ 6.15 (1H, m, NH), 5.77 (1H, s, H-12), 4.89 (1H, br s, H-23), 4.69 (1H, br s, H-23), 4.62 (1H, m, -NCH(CH_3_)-), 3.76 (3H, s, COOCH_3_), 3.61(3H, s, COOCH_3_), 1.75 (3H, s), 1.39 (6H, m), 1.17 (3H, s), 1.16 (3H, s), 1.14 (3H, s), 0.82 (3H, s). ^13^C NMR (100 MHz, CDCl_3_): δ 199.7 (C11), 175.4, 174.5, 173.7, 169.5, 146.7, 128.6 (C12), 114.3 (C23), 53.0, 52.7, 51.7, 51.0, 47.9 (2), 45.2, 43.8, 43.7, 42.1, 38.9, 37.5, 34.6, 32.0, 31.6, 31.5, 29.5, 29.4, 28.6, 26.7, 26.5, 24.0, 23.6, 23.4, 19.7, 18.8, 18.6. ESI-MS m/z: 570.36 (17%), 584.36 ([M + H] ^+^, 100%). Found C 71.09, H 9.51, N 2.35, calcd for C_35_H_53_NO_6_·0.25H_2_O: C 71.46, H 9.17, N 2.38%.

*Methyl 3,4-seco-30-N-methylalaninamido-olean-4(23),12-dien-3-oate***(17)**: To a solution of compound **13** (290 mg, 0.60 mmol) and alanine methyl ester hydrochloride (251 mg, 1.80 mmol) in dichloromethane (8 mL), triethylamine (0.33 mL, 2.40 mmol) was added and the reaction mixture was stirred, at room temperature, for 10 h, after which additional dichloromethane (2 mL) and triethylamine (0.17 mL, 1.20 mmol) and alanine methyl ester hydrochloride (84 mg, 0.60 mmol) were added. After 24 h, the reaction was completed. The work-up was performed as described for compound **15**. The solid was purified by FCC with petroleum ether/ ethyl acetate (3:2) to afford compound **17** as a white solid (48%). m.p.: 100–102 °C. ^1^H NMR (400 MHz, CDCl_3_): δ 6.22 (1H, m, NH), 5.37 (1H, m, H-12), 4.87 (1H, br s, H-23), 4.67 (1H, br s, H-23), 4.63 (1H, m, -NCH(CH_3_)-), 3.76 (3H, s, COOCH_3_), 3.65 (3H, s, COOCH_3_), 1.75 (3H, s), 1.40 (3H, m, -NCH(CH_3_)-), 1.17 (3H, s), 1.11 (3H, s), 1.01 (3H, s), 0.94 (3H, s), 0.79 (3H, s). ^13^C NMR (100 MHz, CDCl_3_): δ 176.2, 174.7, 173.9, 147.6, 144.5, 122.7 (C12), 113.7 (C23), 52.6, 51.7, 50.6, 48.0, 47.9, 44.0, 43.6, 42.2, 39.7, 39.3, 38.1, 38.0, 34.2, 32.2, 31.6, 31.5, 29.8, 28.6, 28.3, 27.0, 26.2, 25.9, 24.6, 23.9, 23.6, 19.7, 18.7, 17.0. ESI-MS m/z: 570.33 ([M + H] ^+^, 100%). Found C 73.04, H 9.94, N 2.45, calcd for C_35_H_55_NO_5_·0.25H_2_O: C 73.20, H 9.74, N 2.44%.

*Methyl 3,4-seco-3-N-glycinamido-11-oxo-olean-4(23),12-dien-30-oate***(18)**: To a solution of compound **6** (120 mg, 0.21 mmol) in methanol (1 mL) and tetrahydrofuran (THF) (1.5 mL), KOH 4N (0.53 ml, 2.10 mmol) was added. After 10 min under magnetic stirring at room temperature, the reaction was completed. The pH of the reaction mixture was neutralized with 10% aqueous HCl. Dichloromethane (30 mL) and water (20 mL) were added to the mixture. The aqueous phase was further extracted with dichloromethane (2 × 30 mL). The combined organic phase was washed with water (2 × 30 mL) and brine (30 mL), dried over Na_2_SO_4_, filtered and the solvent was removed under reduced pressure to afford compound **18** as a white solid (98%). m.p.: 223–225 °C. ^1^H NMR (400 MHz, CDCl_3_): δ 6.76 (1H, m, NH), 5.70 (1H, s, H-12), 4.93 (1H, br s, H-23), 4.78 (1H, br s, H-23), 3.88-4.25 (2H, m, NCH_2_), 3.70 (3H, s, COOCH_3_), 1.80 (3H, s), 1.36 (3H, s), 1.17 (3H, s), 1.15 (3H, s), 1.14 (3H, s), 0.81 (3H, s). ^13^C NMR (100 MHz, CDCl_3_): δ 202.2 (C11), 177.0, 174.5, 173.3, 172.3, 146.5, 127.7 (C12), 114.8 (C23), 53.3, 52.0, 50.8, 48.6, 45.4, 44.2, 44.1, 41.8, 41.4, 39.2, 37.8, 36.1, 32.0, 31.9, 31.5, 31.3, 28.8, 28.3, 26.7, 26.5, 23.9, 23.8, 23.4, 19.7, 18.7. ESI-MS m/z: 556.26 ([M + H] ^+^, 100%).

*Methyl 3,4-seco-3-N-alaninamido-11-oxo-olean-4(23),12-dien-30-oate***(19)**: Compound **19** was prepared using the same method as for the preparation of **18**, but using compound **7** (116 mg, 0.20 mmol), methanol (1 mL), THF (1.5 mL) and KOH 4N (0.50 mL, 2.00 mmol), at room temperature for 10 min, to afford a white solid (94%). m.p.: 216–218 °C. ^1^H NMR (400 MHz, CDCl_3_): δ 6.89 (1H, m, NH), 5.69 (1H, s, H-12), 4.94 (1H, br s, H-23), 4.78 (1H, br s, H-23), 4.61 (1H, m, -NH(CH_3_)-), 3.69 (3H, s, COOCH_3_), 1.80 (3H, s), 1.42 (3H, m, -NH(CH_3_)-), 1.35 (3H, s), 1.16 (3H, s), 1.14 (6H, s), 0.81 (3H, s). ^13^C NMR (100 MHz, CDCl_3_): δ 202.0 (C11), 176.8, 175.3, 174.0, 173.1, 146.3, 127.6 (C12), 114.6 (C23), 53.2, 51.9, 50.6, 48.5, 48.0, 45.3, 44.0 (2), 41.2, 39.0, 37.7, 36.1, 31.9, 31.8, 31.3, 31.1, 28.6, 28.1, 26.5, 26.4, 23.8 (2), 23.2, 19.5, 18.7, 18.6. ESI-MS m/z: 570.26 ([M + H] ^+^, 100%).

*3,4-seco-30-N-glycinamido-11-oxo-olean-4(23),12-dien-3-oic acid***(20)**: The method followed that of compound **18**, using compound **14** (145 mg, 0.26 mmol), methanol (1 mL), THF (1.5 mL) and KOH 4N (0.65 mL, 2.60 mmol), at room temperature for 10 min, to afford compound **20** as a white solid (97%). m.p.: 213–215 °C. ^1^H NMR (400 MHz, CDCl_3_): δ 7.58 (1H, m, NH), 5.77 (1H, s, H-12), 4.88 (1H, br s, H-23), 4.62 (1H, br s, H-23), 3.50-4.48 (2H, m, NCH_2_), 1.72 (3H, s), 1.42 (3H, s), 1.21 (3H, s), 1.16 (3H, s), 1.09 (3H, s), 0.83 (3H, s). ^13^C NMR (100 MHz, CDCl_3_): δ 202.1 (C11), 181.0, 177.3, 173.1, 172.7, 146.5, 128.0 (C12), 114.5 (C23), 52.5, 50.6, 47.5, 45.7, 44.2, 44.0, 42.1, 41.1, 39.5, 37.5, 34.5, 31.8, 31.6 (2), 30.2, 29.1, 28.7, 26.8 (2), 23.7, 23.4, 22.9, 19.8, 18.8. ESI-MS m/z: 542.26 ([M + H] ^+^, 100%).

*3,4-seco-30-N-alaninamido-11-oxo-olean-4(23),12-dien-3-oic acid***(21)**: Compound **21** was prepared using the same method as for the preparation of **18**, but using compound **16** (170 mg, 0.29 mmol), methanol (1 mL), THF (1.5 mL) and KOH 4N (0.73 mL, 2.90 mmol), at room temperature for 10 min, to afford a white solid (98%). m.p.: 224–226 °C. ^1^H NMR (400 MHz, CDCl_3_): δ 7.37 (1H, m, NH), 5.74 (1H, s, H-12), 4.88 (1H, br s, H-23), 4.78 (1H, m, -NH(CH_3_)-), 4.68 (1H, br s, H-23), 1.74 (3H, s), 1.15-1.35 (15H, m), 0.82 (3H, s). ^13^C NMR (100 MHz, CDCl_3_): δ 201.1 (C11), 179.8, 176.9, 176.3, 171.8, 146.6, 128.1 (C12), 114.5 (C23), 52.7, 50.7, 47.8, 47.7, 45.7, 44.0, 43.8, 41.9, 39.2, 37.6, 34.1, 31.9, 31.6 (2), 29.6, 29.1, 28.7, 26.8, 26.7, 23.8, 23.5, 23.2, 19.8, 18.8, 17.6. ESI-MS m/z: 272.01 (10%), 527.35 (12), 556.25 ([M + H] ^+^, 100%).

### 3.2. Biology

A549, HT-29, Jurkat, MOLT-4, MIA Paca 2, MCF7, HeLa, A375, HepG2, and BJ cells were obtained from the American Type Culture Collection (ATCC, Rockville, MD, USA). Dulbecco´s Modified Eagle Medium (DMEM), Roswell Park Memorial Institute (RPMI)-1640 medium, Phosphate Buffered Saline (PBS), glucose 45%, human insulin 10 mg/mL, dimethyl sulfoxide (DMSO), 3-(4,5-dimethylthiazol-2-yl)-2,5-diphenyl tetrazolium bromide (MTT) powder, Trypan Blue (TB) 0.4%, propidium iodide (PI) and Hoescht 33342 were purchased from Sigma Aldrich Co. (St Louis, MO, USA). Minimum Essential Medium (MEM), penicillin/streptomycin (P/S) and l-glutamine were purchased from Gibco-BRL (Eggenstein, Germany). Sodium pyruvate, trypsin/EDTA (0.05%/0.02%) and MEM-Eagle Non-Essential Aminoacids 100× were obtained from Biological Industries (Kibbutz Beit Haemek, Israel). Fetal Bovine Serum (FBS) was obtained from PAA Laboratories (Pasching, Austria), the Cell Proliferation Kit II (XTT kit) was purchased from Roche (Roche Molecular Biochemicals, Indianapolis, IN, USA) and annexin V-FITC was obtained from Bender MedSystems (Vienna, Austria).

Stock solutions of 20 mM in DMSO of the synthesized compounds were prepared and stored at −20 °C. Working solutions were prepared in culture medium and appropriate amounts of DMSO were included in controls; all solutions had a final concentration of 0.5% DMSO.

#### 3.2.1. Cell Culture

A549, HT-29, MIA Paca 2, HeLa and A375 cells were cultured in DMEM supplemented with 10% heat-inactivated FBS and 1% P/S. HepG2 and BJ cells were grown in DMEM supplemented with 10% heat-inactivated FBS, 1% P/S and 1mM sodium pyruvate. Jurkat and MOLT-4 cells were cultured in RPMI-1640 medium supplemented with 10% heat-inactivated FBS, 1% P/S and 2 mM l-glutamine. MCF7 cells were maintained in MEM supplemented with 10% heat-inactivated FBS, 0.1% P/S, 1mM sodium pyruvate, 2 mM l-glutamine, 1× MEM-Eagle Non-Essential Aminoacids, 0.01 mg/mL insulin human and 10 mM glucose. 

All cell cultures were performed at 37 °C in an atmosphere of 5% CO_2_.

#### 3.2.2. Antiproliferative Activity Assays

The antiproliferative activity of the synthesized compounds on A549, HT-29, MIA Paca 2, MCF7, HeLa, A375, HepG2 and BJ adherent cells was determined by the MTT assay. Exponentially growing cells were plated in 96-well plates at a density of 1–8 × 10^3^ cells/ well. After 24 h, cells were attached to the plate, and the growth medium was replaced with fresh medium containing either the compounds dissolved in DMSO at different concentrations or only DMSO, in triplicate, and the cells were continued to culture for 72 h. After incubation with the compounds, the medium was removed and 100 µL of MTT solution (0.5 mg/mL) were added to each well and the plates were incubated for 1 h. MTT was removed and 100 µL of DMSO was added to dissolve the formazan crystals. The absorbance was immediately read at 550 nm on an ELISA read plater (Tecan Sunrise MR20-301, TECAN, Austria). For Jurkat and MOLT-4 non-adherent cells, the antiproliferative activity was determined by XTT assay. These cell lines were plated with 5.5 × 10^3^ and 1 × 10^4^ cells/well, respectively, in 96-well plates in 100 µL medium. The seeding was executed simultaneously with the addition of the different concentrations of compounds or vehicle, in triplicate, and cells were allowed to incubate for 72 h. After that incubation period, 100 μL of the XTT labelling mixture were added to each well and the plates were incubated again for 4 h. Then, the absorbance was read at 450 nm on the ELISA plate reader. 

Concentrations that inhibit cell proliferation by 50% (IC_50_) represent an average of a minimum of three independent experiments and were expressed as means ± standard deviation (SD).

#### 3.2.3. Cell Cycle Analysis

Cell cycle was assessed by flow cytometry using a fluorescence-activated cell sorter (FACS). Jurkat cells were plated in six-well plates at a density of 1.6 × 10^5^ cells/well, simultaneously with the addition of compound **17**, at a concentration corresponding to its IC_50_ value at 72 h of treatment, or with only the vehicle, in a total volume of 2 mL of medium. The cells were allowed to incubate for 24, 48 and 72 h. After incubation, cells were collected and centrifuged. The supernatant was removed and the pellet was resuspended in 1 mL of TBS containing 1 mg/mL PI, 10 mg/mL RNase free of DNase and 0.1% Igepal CA-630, for 1 h, at 4 °C. FACS analysis was performed at 488 nm in an Epics XL flow cytometer (Coulter Corporation, Hialeah, FL, USA). Data were collected and analyzed using the Multicycle software (Phoenix Flow Systems, San Diego, CA, USA). Three independent experiments were performed, with two replicates per experiment.

#### 3.2.4. Annexin V-FITC/PI Flow Cytometry Assay

Apoptosis was assessed by flow cytometry using a FACS. Jurkat cells were plated in six-well plates at a density of 1.6 × 10^5^ cells/well, simultaneously with the addition of compound **17**, at specified concentrations, or with only the vehicle, in a total volume of 2 mL of medium. The cells were allowed to incubate for 24, 48 and 72 h. After incubation, cells were collected and centrifuged. The supernatant was removed and the pellet was resuspended in 95 µL of binding buffer (10 mM HEPES/NaOH, pH 7.4, 140 mM NaCl, 2.5 mM CaCl_2_). Annexin V-FITC conjugate (3 µL) was added and cells were incubated for 30 min, at room temperature, in darkness. After incubation, 0.8 mL of binding buffer were added. Just before the FACS analysis, cells were stained with 20 µL of 1 mg/mL PI solution. Three independent experiments were performed, with two replicates per experiment.

#### 3.2.5. Hoechst 33342 Staining

The morphological changes were observed by fluorescence microscopy using Hoechst staining. Jurkat cells were plated in six-well plates at a density of 1.6 × 10^5^ cells/well, simultaneously with the addition of compound **17**, at specified concentrations, or with only the vehicle, in a total volume of 2 mL of medium. The cells were incubated for 72 h. After incubation, cells were collected by centrifugation, washed twice with PBS and stained with 500 µL of Hoechst 33342 solution (2 µg/ml in PBS), for 15 min, at room temperature, in darkness. Finally, cells were washed and resuspended in 10 µL PBS. The samples were mounted on a slide and observed with a fluorescence microscope (DMRB, Leica Microssystems, Wetzlar, Germany) with a 4′,6-diamidine-2′-phenylindole dihydrochloride (DAPI) filter. Three independent experiments were conducted.

## 4. Conclusions

In summary, we synthesized a series of new GA derivatives via the opening of its ring A along with the coupling of an amino acid. Antiproliferative activity assays in a panel of nine human cancer cell lines showed that the most potent compound **17** was 5 to 17-fold more active than **GA 1**. The study of selectivity revealed that this new derivative was up to 10 times more selective towards malignant cells than its parental compound. Preliminary mechanism investigation indicated that compound **17** may act through arresting cell cycle progression at the S phase and inducing apoptosis. The enhanced potency and the high selectivity of this new GA derivative warrant further biological evaluation.

## Figures and Tables

**Table 1:** Antiproliferative activities of GA **1**, its derivatives **5**–**7** and **10**–**21**, and cisplatin against A549 and HT-29 cell lines.

Compound	Cell line (IC_50_, µM) ^1^	
	A549	HT-29
**1**	110.5 ± 3.9	115.7 ± 1.6
**5**	59.4 ± 2.1	66.6 ± 3.2
**6**	31.6 ± 1.5	37.4 ± 1.0
**7**	26.2 ± 2.4	24.4 ± 1.7
**10**	52.2 ± 3.0	61.7 ± 1.4
**11**	33.7 ± 2.0	43.0 ± 1.5
**12**	> 100	> 100
**13**	> 100	> 100
**14**	33.7 ± 1.8	35.0 ± 1.7
**15**	26.4 ± 2.2	24.7 ± 0.9
**16**	24.4 ± 1.4	23.8 ± 0.3
**17**	14.8 ± 0.9	13.0 ± 0.5
**18**	> 100	> 100
**19**	> 100	> 100
**20**	> 100	> 100
**21**	> 100	> 100
**Cisplatin**	12.6 ± 0.8 [48]	6.1 [49]

^1^ The cell lines were treated with different concentrations of each compound for 72 h. IC_50_ values were determined by MTT assay and are expressed as means ± SD (standard deviation) of three independent experiments.

**Table 2:** Antiproliferative activities of GA **1**, its derivatives **6**, **7** and **14–21**, and cisplatin against several cancer cell lines and the human nontumoral BJ cell line.

Compound	Cell line (IC_50_, µM) ^1^							
	Jurkat	MOLT-4	MIAPaca2	MCF7	HeLa	A375	HepG2	BJ
**1**	105.6 ± 5.0	95.5 ± 3.9	101.6 ± 1.6	97.8 ± 3.9	107.2 ± 2.5	112.2 ± 2.6	125.1 ± 9.1	165.0 ± 7.1
**6**	11.9 ± 0.2	18.9 ± 1.6	28.2 ± 0.5	32.9 ± 1.6	34.5 ± 2.5	30.0 ± 1.5	30.6 ± 0.5	N.D.
**7**	11.7 ± 0.6	18.5 ± 0.9	24.9 ± 1.2	24.9 ± 0.9	25.7 ± 0.6	24.5 ± 1.0	24.8 ± 0.4	N.D.
**14**	13.3 ± 1.1	23.5 ± 0.8	32.5 ± 3.2	28.8 ± 0.7	34.2 ± 2.4	30.0 ± 2.2	34.7 ± 1.1	N.D.
**15**	12.5 ± 0.5	18.9 ± 1.6	20.2 ± 1.2	24.8 ± 1.3	22.2 ± 0.3	18.8 ± 1.1	25.4 ± 1.3	N.D.
**16**	9.6 ± 0.4	19.1 ± 1.3	22.6 ± 0.6	23.8 ± 1.6	19.1 ± 0.5	17.0 ± 1.1	25.7 ± 0.8	N.D.
**17**	6.1 ± 0.2	15.3 ± 0.7	11.8 ± 1.1	21.6 ± 0.6	13.0 ± 0.5	11.3 ± 0.4	16.0 ± 0.3	> 100
**18**	46.4 ± 3.7	51.9 ± 2.5	N.D.	N.D.	N.D.	N.D.	N.D.	N.D.
**19**	40.8 ± 2.7	49.0 ± 1.6	N.D.	N.D.	N.D.	N.D.	N.D.	N.D.
**20**	> 100	> 100	N.D.	N.D.	N.D.	N.D.	N.D.	N.D.
**21**	> 100	> 100	N.D.	N.D.	N.D.	N.D.	N.D.	N.D.
**Cisplatin**	1.9 [50]	1.4 [50]	5.0 ± 1.0 [51]	19.1 ± 4.5 [52]	2.3 ± 0.3 [52]	3.1 ± 1.0 [48]	2.9 [49]	10.1 ± 2.0 [52]

^1^ The cell lines were treated with different concentrations of each compound for 72 h. IC_50_ values were determined by XTT assay in Jurkat and MOLT-4 cells and by MTT assay in all the other cell lines. Results are expressed as means ± SD of three independent experiments. N.D.: not determined.

## Supplementary Materials

^1^H-NMR and ^13^C-NMR spectra of selected compounds are available online.
