# Supplementary material for: Synthesis and Antiproliferative Activity of Novel A-Ring Cleaved Glycyrrhetinic Acid Derivatives
Source: Molecules. 2019 Aug 14;24(16):2938. doi: 10.3390/molecules24162938 (PMC6721064; doi:10.3390/molecules24162938)

Supplementary information

Synthesis and Antiproliferative Activity of Novel A-Ring Cleaved Glycyrrhetinic Acid Derivatives

Daniela P. S. Alho [1,2], Jorge A. R. Salvador [1,2,*], Marta Cascante [3,4] and Silvia Marín [3,4,*]

[1] Laboratory of Pharmaceutical Chemistry, Faculty of Pharmacy, University of Coimbra, 3000-548 Coimbra, Portugal.

[2] Center of Neuroscience and Cell Biology, 3000- 504 Coimbra, Portugal.

[3] Department of Biochemistry and Molecular Biomedicine, Faculty of Biology, University of Barcelona, Diagonal 643, 08028 Barcelona.

[4] Centro de Investigación Biomédica en Red de Enfermedades Hepáticas y Digestivas (CIBEREHD), Instituto de Salud Carlos III (ISCIII), 28029 Madrid, Spain.

Table of contents

1. NMR data for compound 5 4
   1.1. ^{1}H NMR spectrum for compound 5 recorded in $CDCl_3$ 4
   1.2. ^{13}C NMR spectrum for compound 5 recorded in $CDCl_3$ 5
   1.3. DEPT-135 spectrum for compound 5 recorded in $CDCl_3$ 6
2. NMR data for compound 6 7
   2.1. ^{1}H NMR spectrum for compound 6 recorded in $CDCl_3$ 7
   2.2. ^{13}C NMR spectrum for compound 6 recorded in $CDCl_3$ 8
   2.3. DEPT-135 spectrum for compound 6 recorded in $CDCl_3$ 9
3. NMR data for compound 7 10
   3.1. ^{1}H NMR spectrum for compound 7 recorded in $CDCl_3$ 10
   3.2. ^{13}C NMR spectrum for compound 7 recorded in $CDCl_3$ 11
   3.3. DEPT-135 spectrum for compound 7 recorded in $CDCl_3$ 12
4. NMR data for compound 10 13
   4.1. ^{1}H NMR spectrum for compound 10 recorded in $CDCl_3$ 13
   4.2. ^{13}C NMR spectrum for compound 10 recorded in $CDCl_3$ 14
   4.3. DEPT-135 spectrum for compound 10 recorded in $CDCl_3$ 15
5. NMR data for compound 11 16
   5.1. ^{1}H NMR spectrum for compound 11 recorded in $CDCl_3$ 16
   5.2. ^{13}C NMR spectrum for compound 11 recorded in $CDCl_3$ 17
   5.3. DEPT-135 spectrum for compound 11 recorded in $CDCl_3$ 18
6. NMR data for compound 12 19
   6.1. ^{1}H NMR spectrum for compound 12 recorded in $CDCl_3$ 19
   6.2. ^{13}C NMR spectrum for compound 12 recorded in $CDCl_3$ 20
   6.3. DEPT-135 spectrum for compound 12 recorded in $CDCl_3$ 21
7. NMR data for compound 14 22
   7.1. ^{1}H NMR spectrum for compound 14 recorded in $CDCl_3$ 22
   7.2. ^{13}C NMR spectrum for compound 14 recorded in $CDCl_3$ 23
   7.3. DEPT-135 spectrum for compound 14 recorded in $CDCl_3$ 24
8. NMR data for compound 17 25
   8.1. ^{1}H NMR spectrum for compound 17 recorded in $CDCl_3$ 25
   8.2. ^{13}C NMR spectrum for compound 17 recorded in $CDCl_3$ 26
   8.3. DEPT-135 spectrum for compound 17 recorded in $CDCl_3$ 27
9. NMR data for compound 18 28
   9.1. ^{1}H NMR spectrum for compound 18 recorded in $CDCl_3$ 28
   9.2. ^{13}C NMR spectrum for compound 18 recorded in $CDCl_3$ 29

9.3. DEPT-135 spectrum for compound 18 recorded in $CDCl_3$ 30
10. NMR data for compound 19 31
10.1. ^{1}H NMR spectrum for compound 19 recorded in $CDCl_3$ 31
10.2. ^{13}C NMR spectrum for compound 19 recorded in $CDCl_3$ 32
10.3. DEPT-135 spectrum for compound 19 recorded in $CDCl_3$ 33
11. NMR data for compound 20 34
11.1. ^{1}H NMR spectrum for compound 20 recorded in $CDCl_3$ 34
11.2. ^{13}C NMR spectrum for compound 20 recorded in $CDCl_3$ 35
11.3. DEPT-135 spectrum for compound 20 recorded in $CDCl_3$ 36

1. NMR data for compound 5

1.1. ^{1}H NMR spectrum for compound 5 recorded in $CDCl_3$

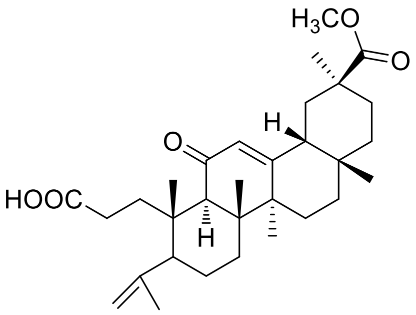

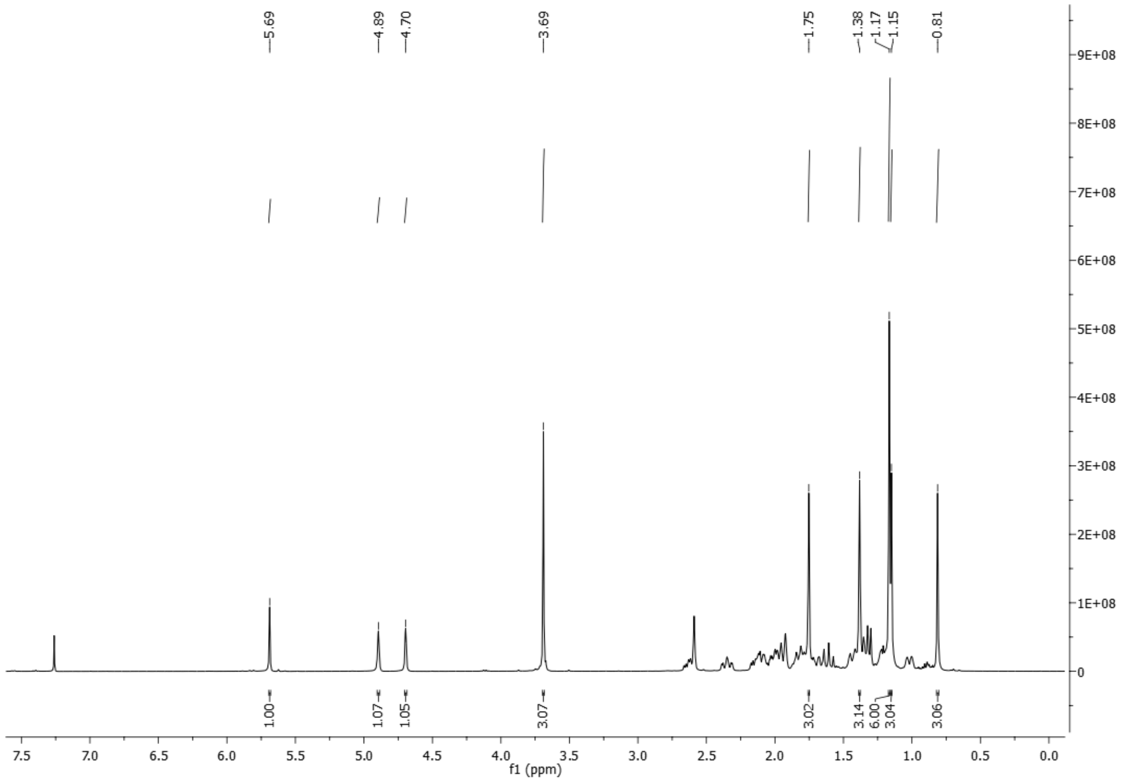

1.2. ^{13}C NMR spectrum for compound 5 recorded in $CDCl_3$

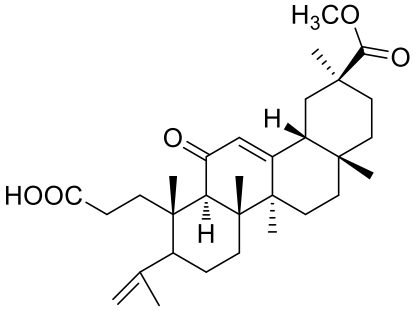

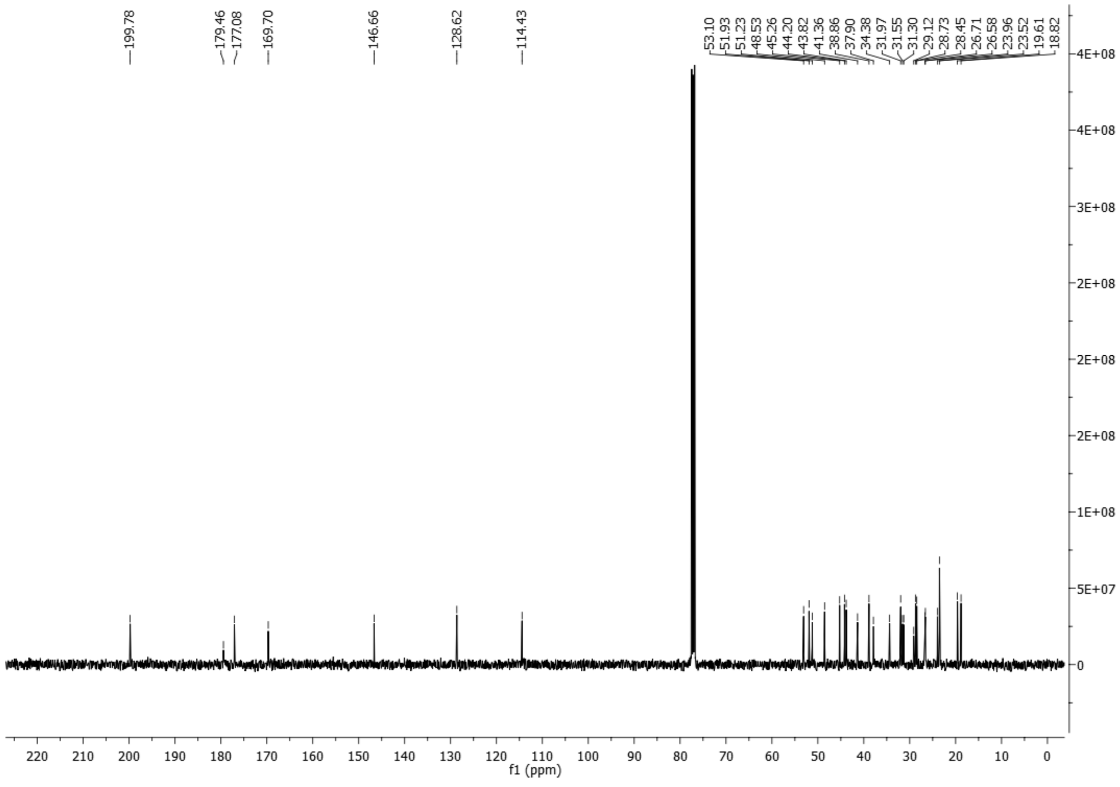

1.3. DEPT-135 spectrum for compound 5 recorded in $CDCl_3$

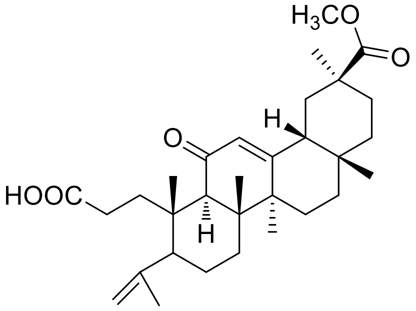

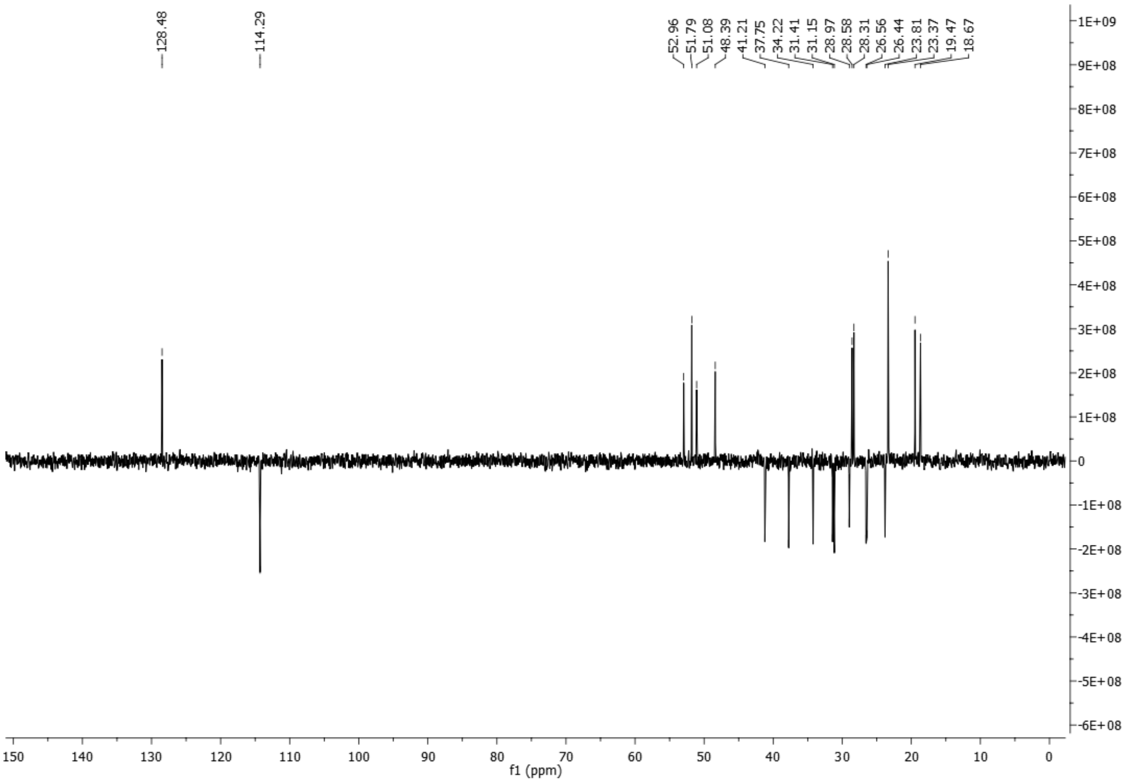

2. NMR data for compound 6

2.1. ^{1}H NMR spectrum for compound 6 recorded in $CDCl_3$

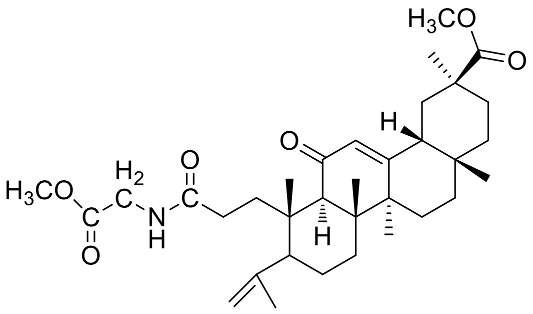

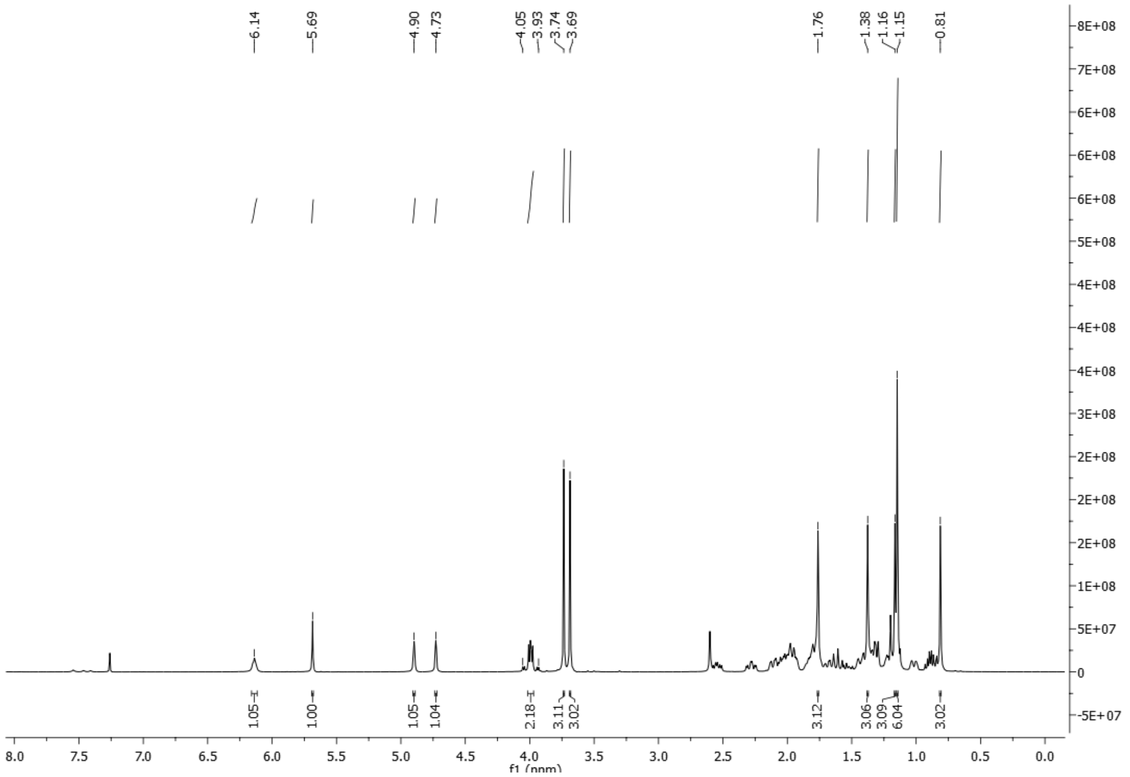

2.2. ^{13}C NMR spectrum for compound 6 recorded in $CDCl_3$

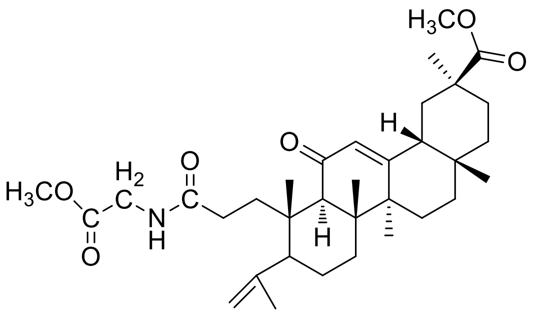

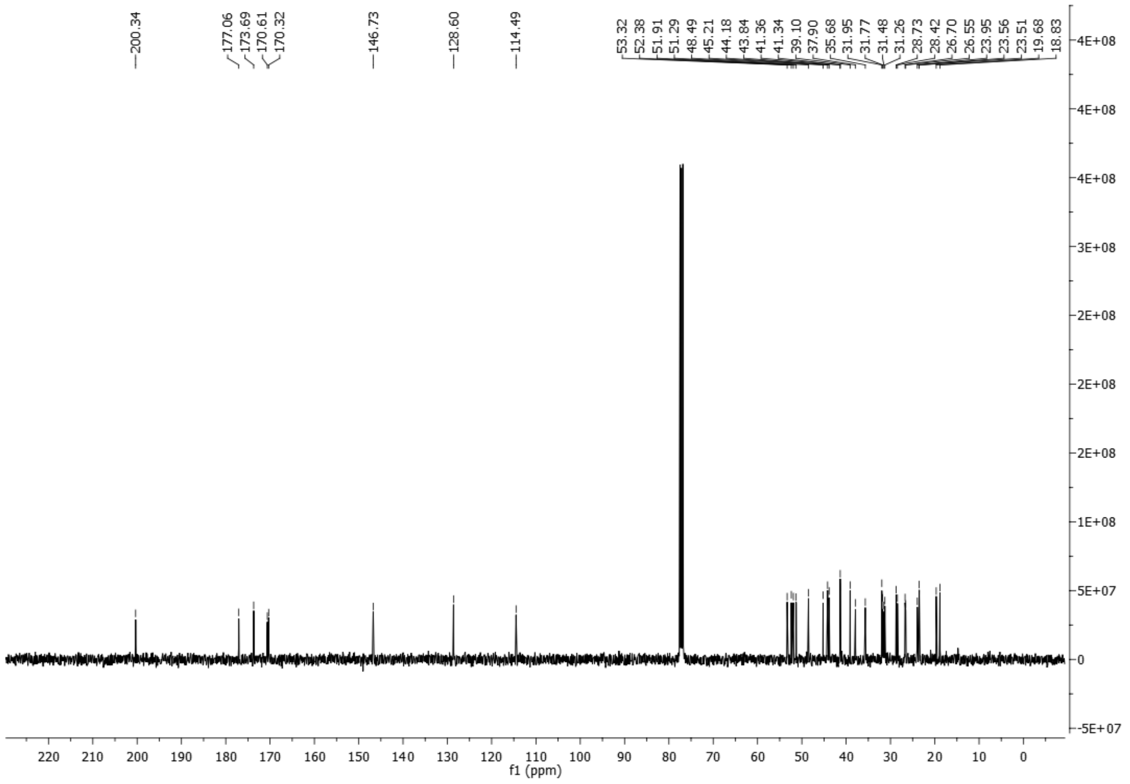

2.3. DEPT-135 spectrum for compound 6 recorded in $CDCl_3$

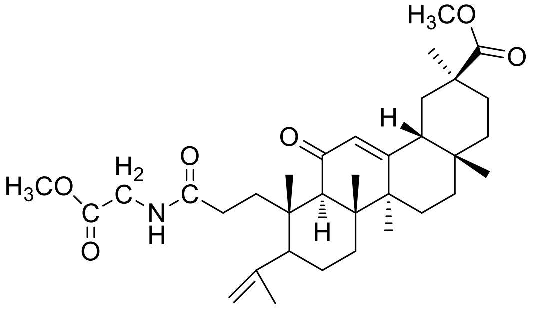

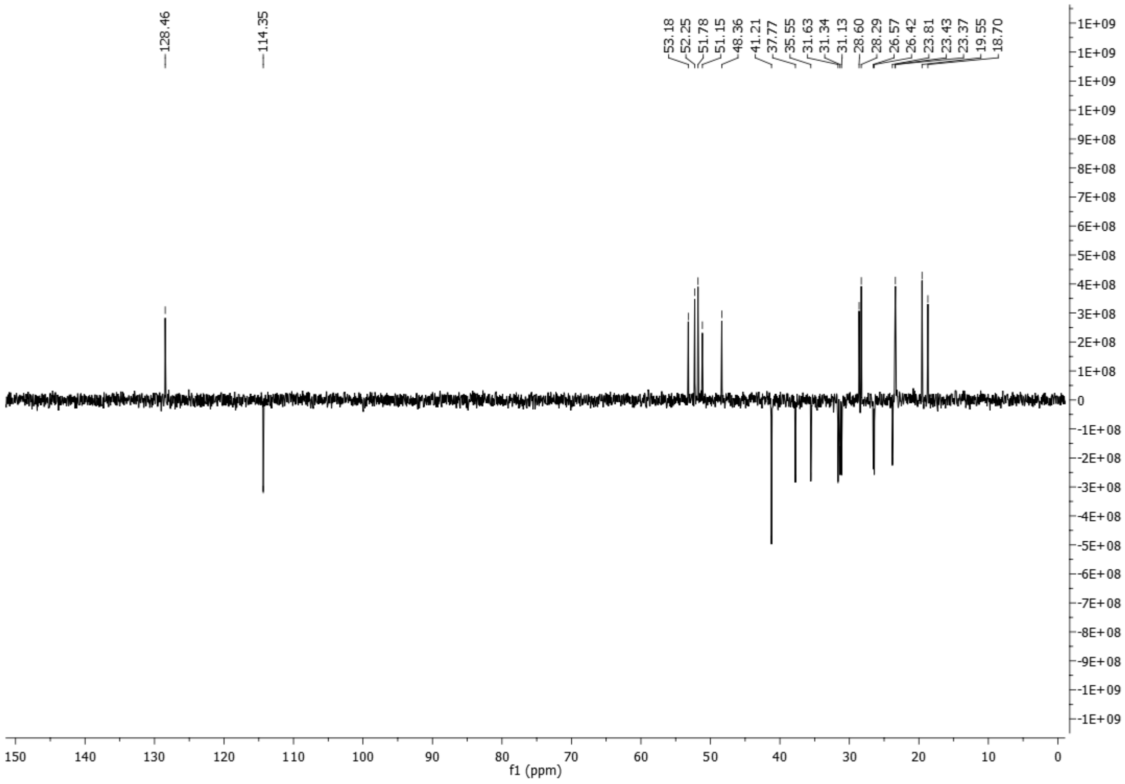

3. NMR data for compound 7

3.1. ^{1}H NMR spectrum for compound 7 recorded in $CDCl_3$

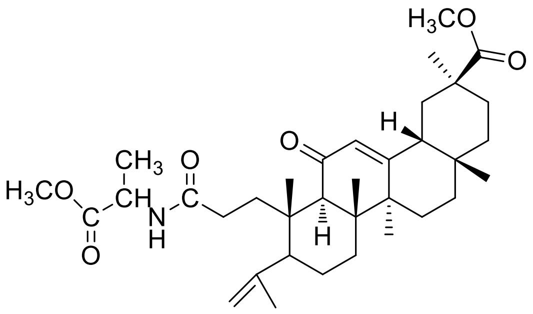

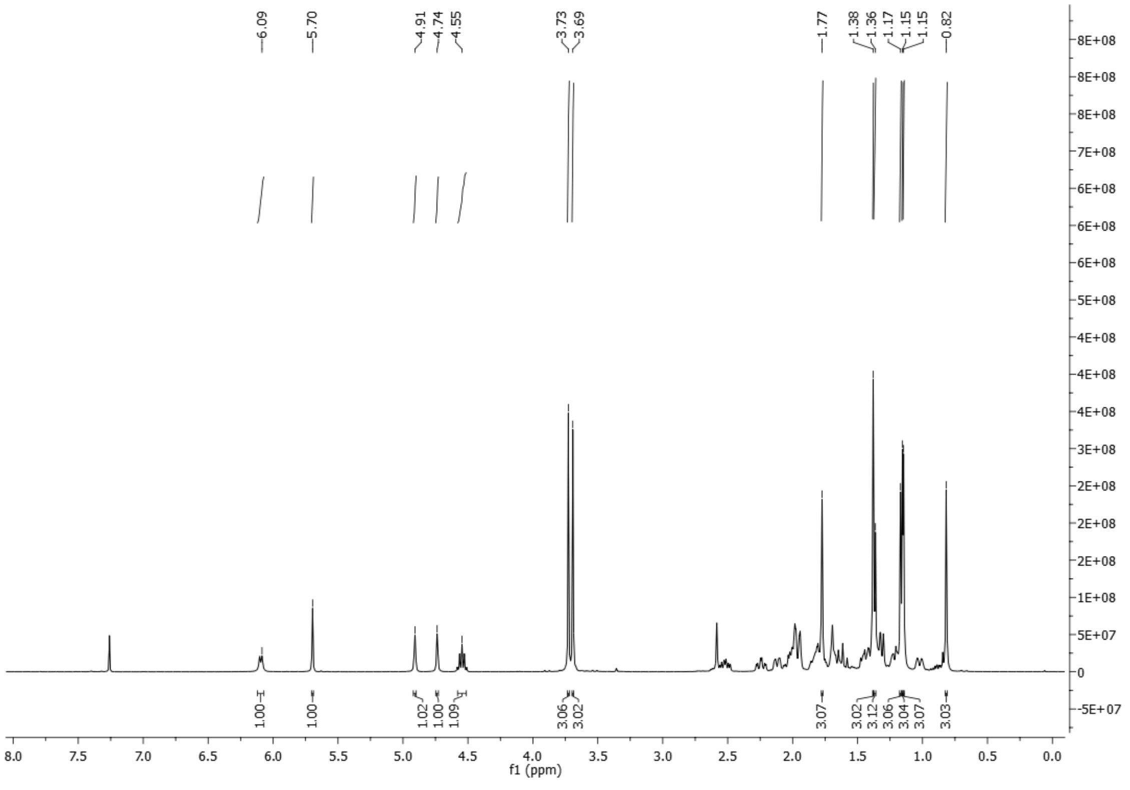

3.2. ^{13}C NMR spectrum for compound 7 recorded in $CDCl_3$

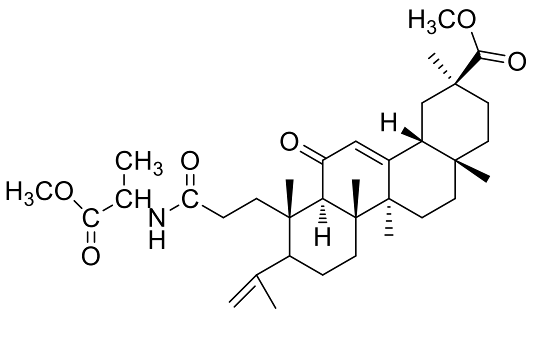

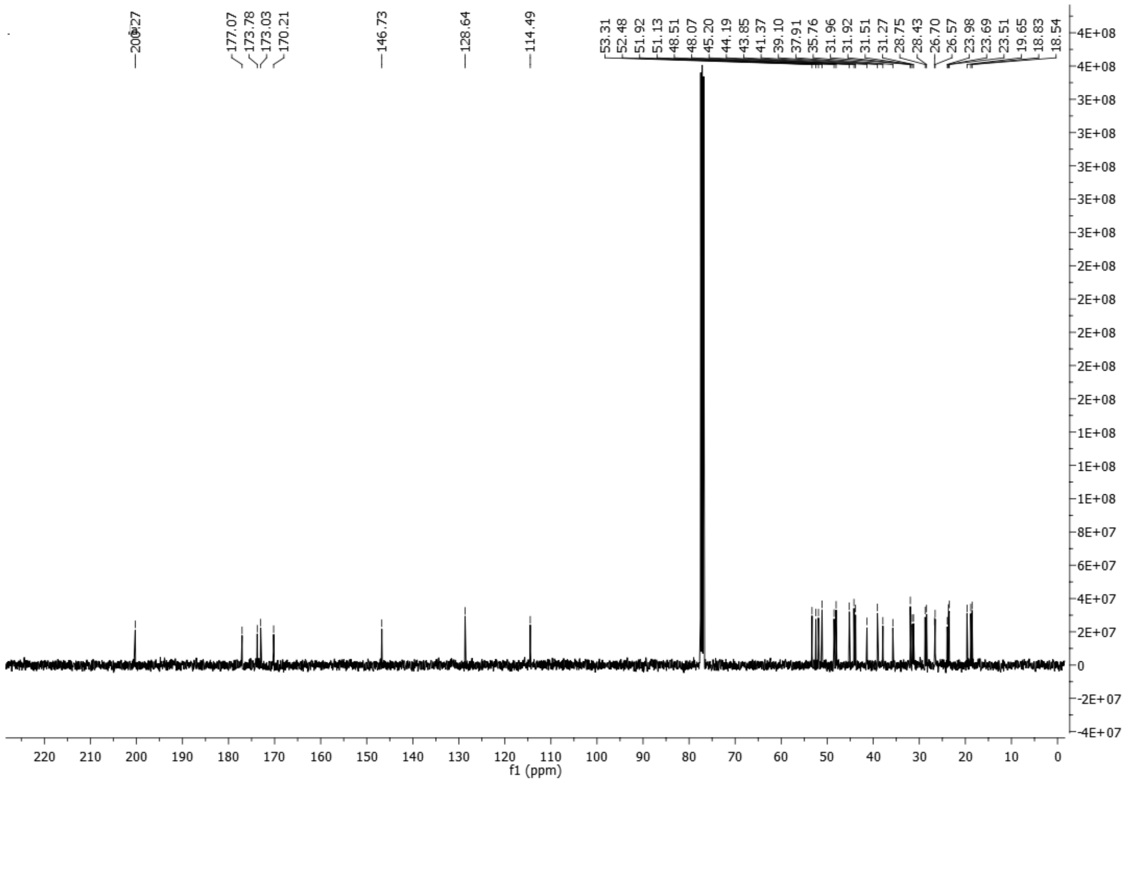

3.3. DEPT-135 spectrum for compound 7 recorded in $CDCl_3$

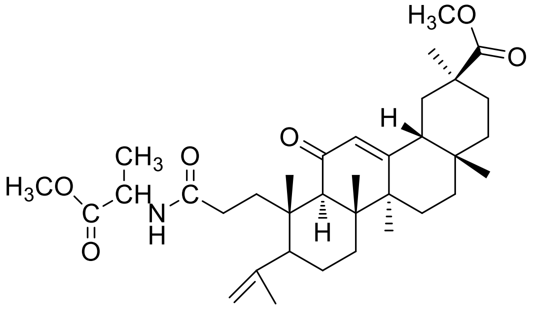

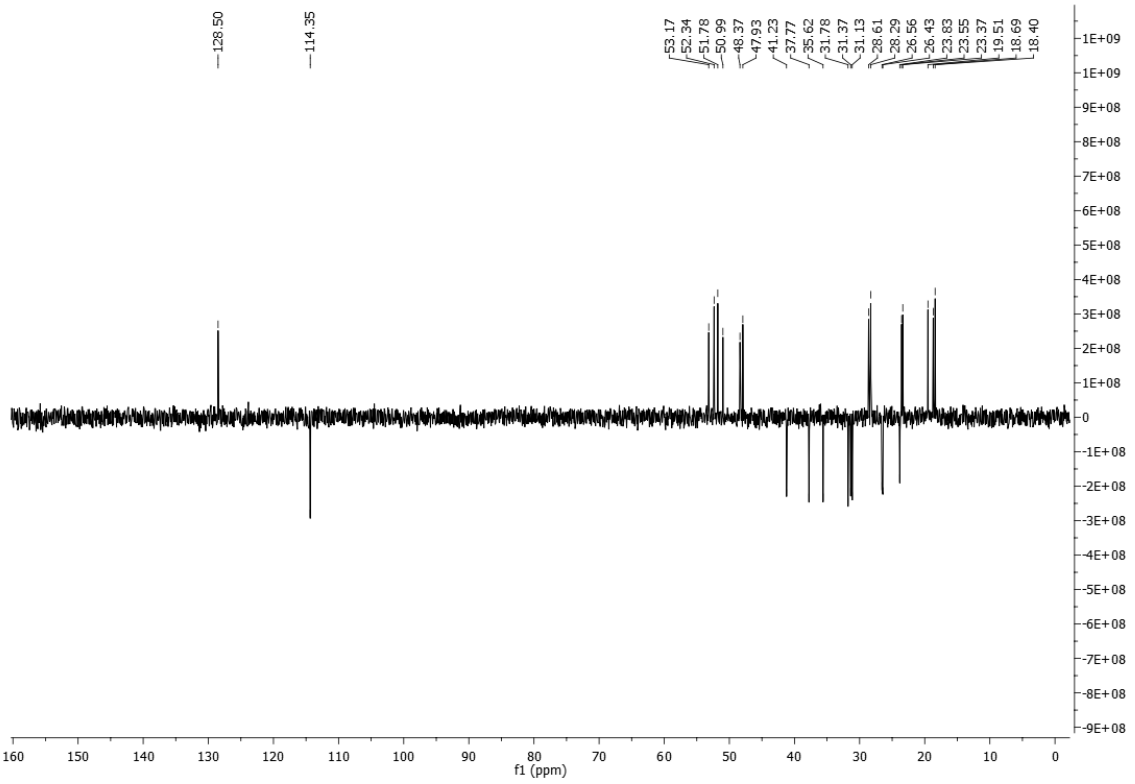

4. NMR data for compound 10

4.1. ^{1}H NMR spectrum for compound 10 recorded in $CDCl_3$

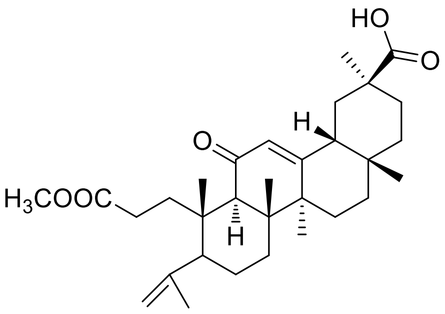

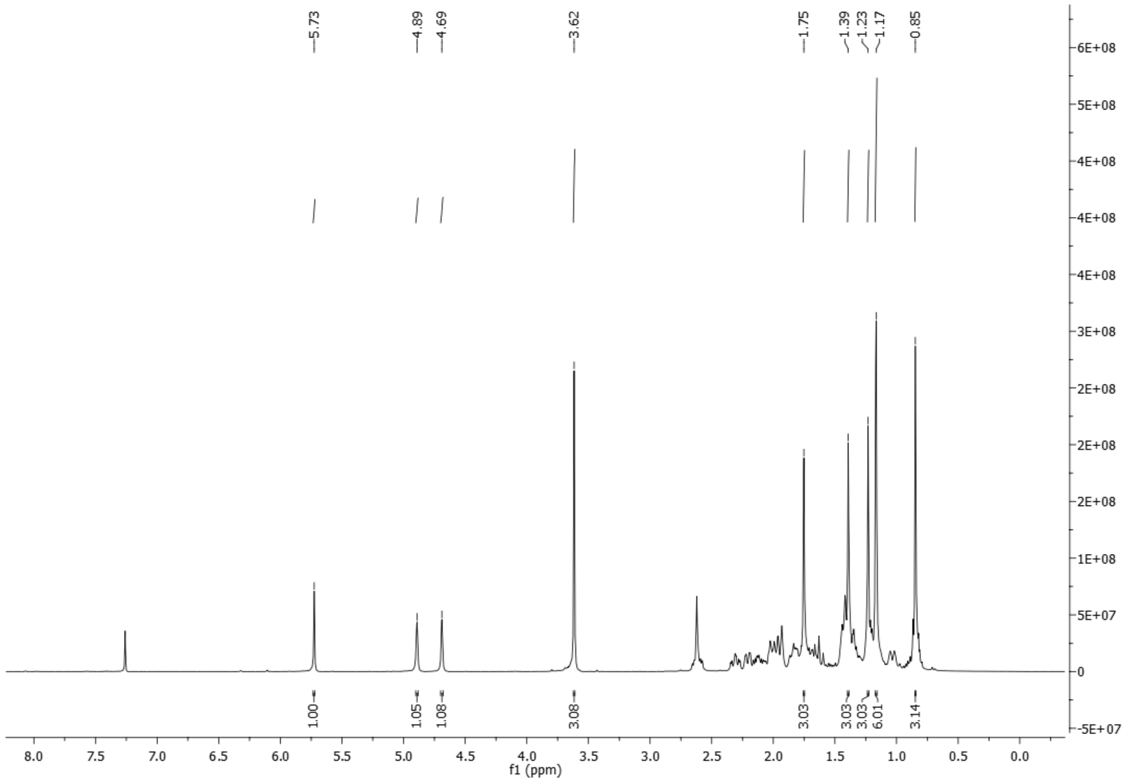

4.2. ^{13}C NMR spectrum for compound 10 recorded in $CDCl_3$

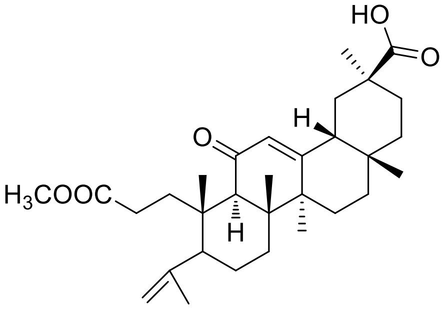

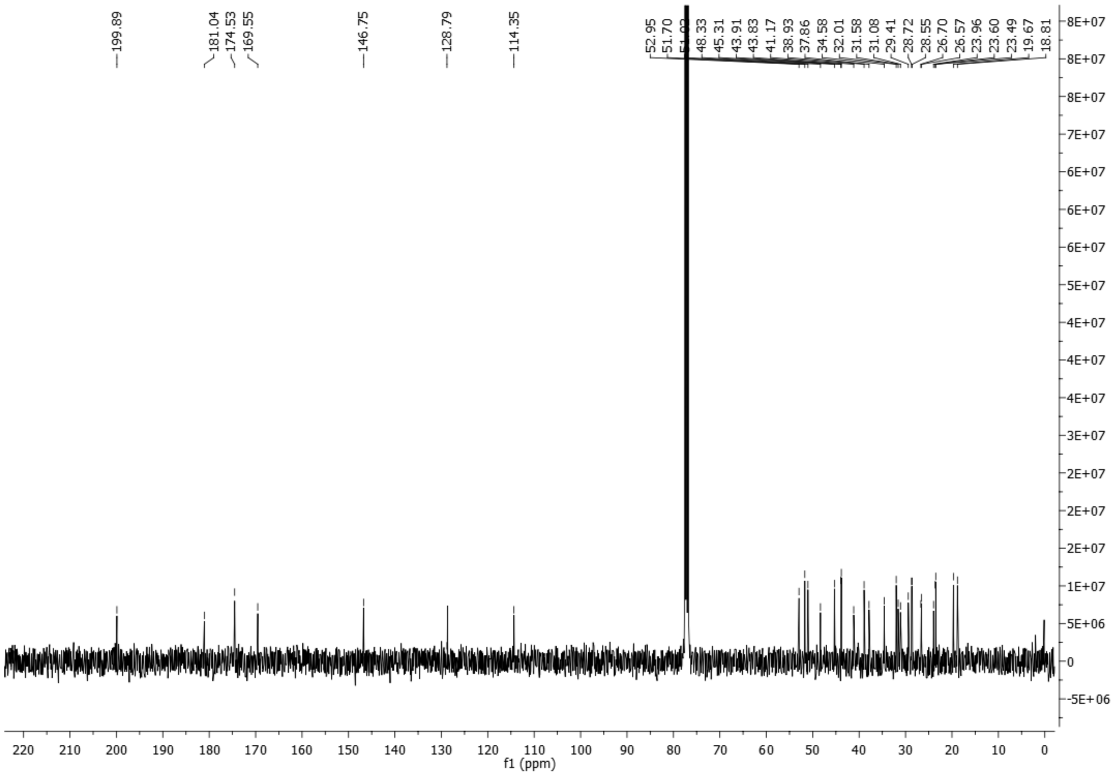

4.3. DEPT-135 spectrum for compound 10 recorded in $CDCl_3$

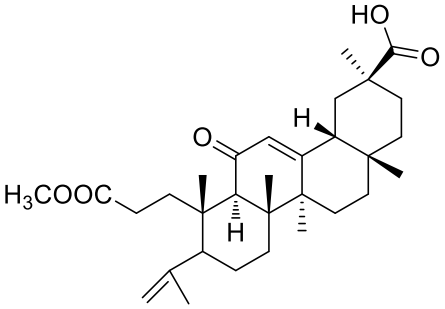

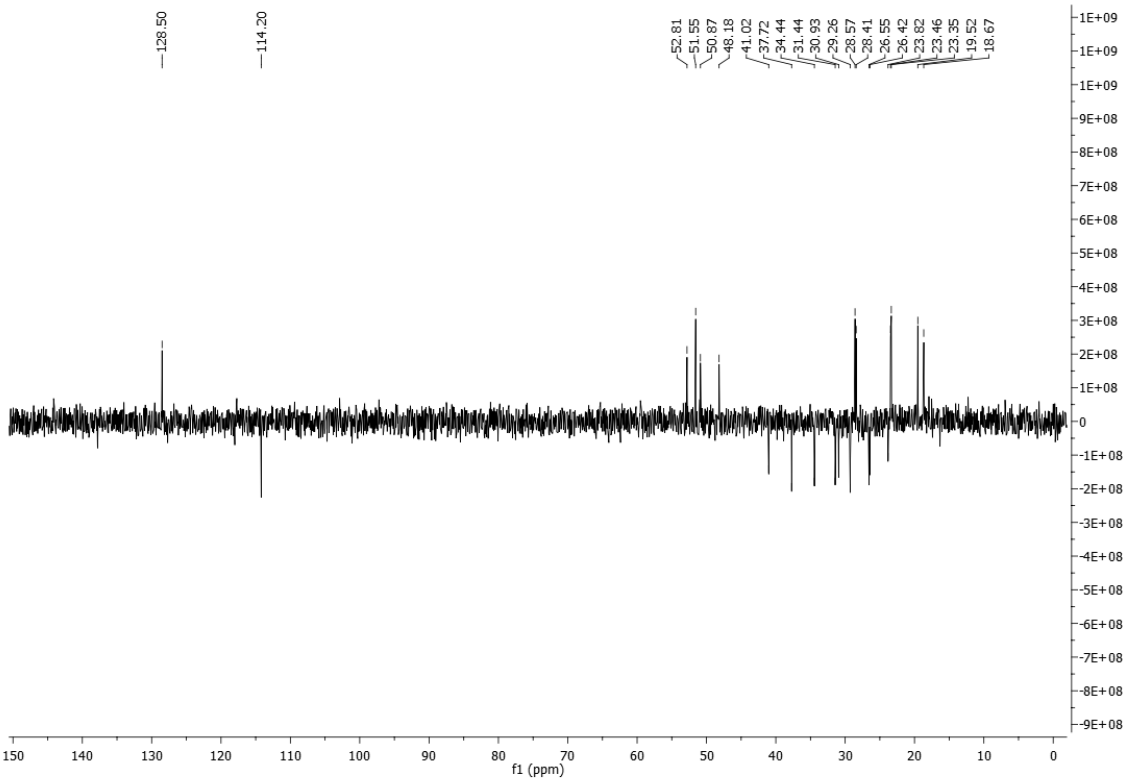

5. NMR data for compound 11

5.1. ^{1}H NMR spectrum for compound 11 recorded in $CDCl_3$

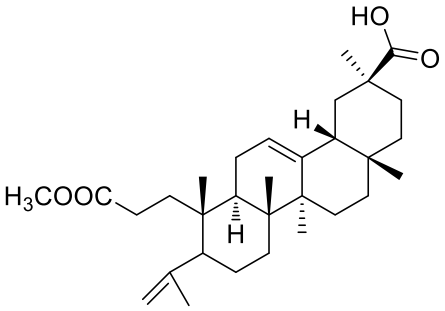

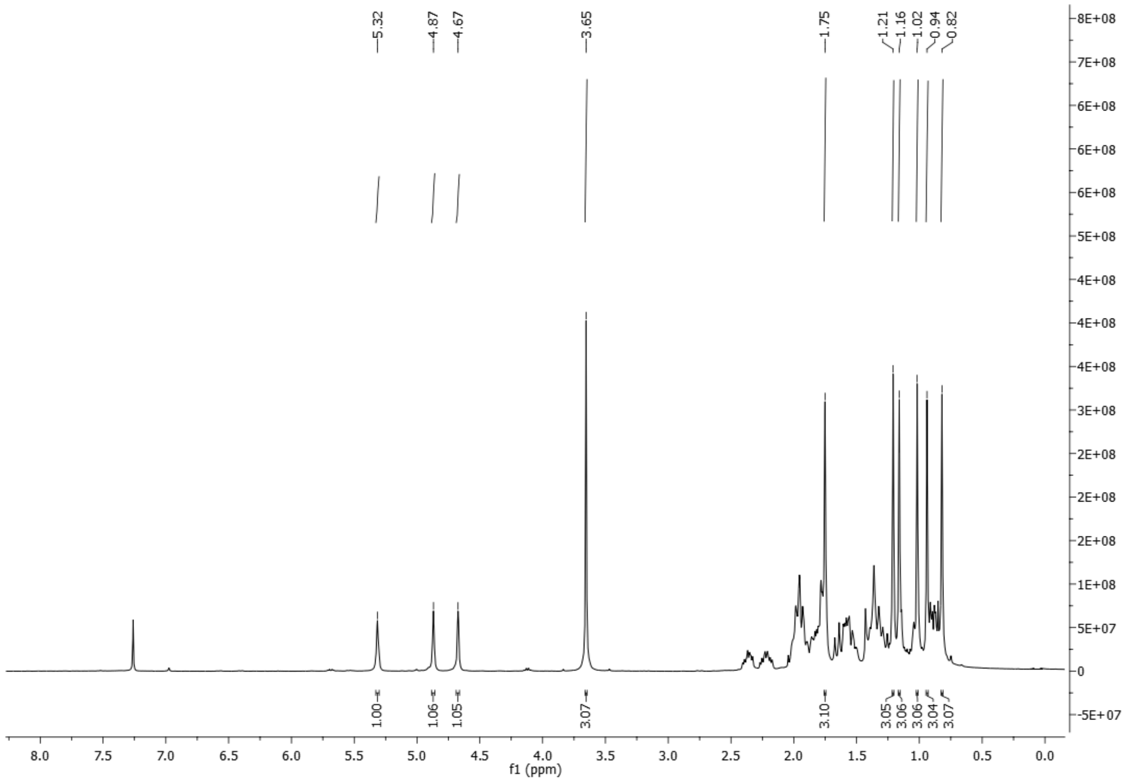

5.2. ^{13}C NMR spectrum for compound 11 recorded in $CDCl_3$

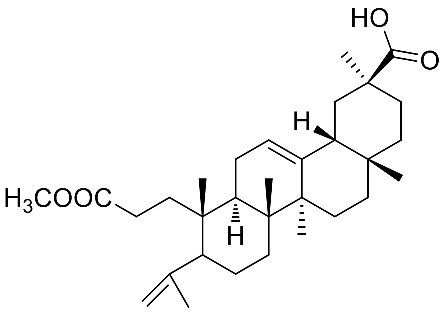

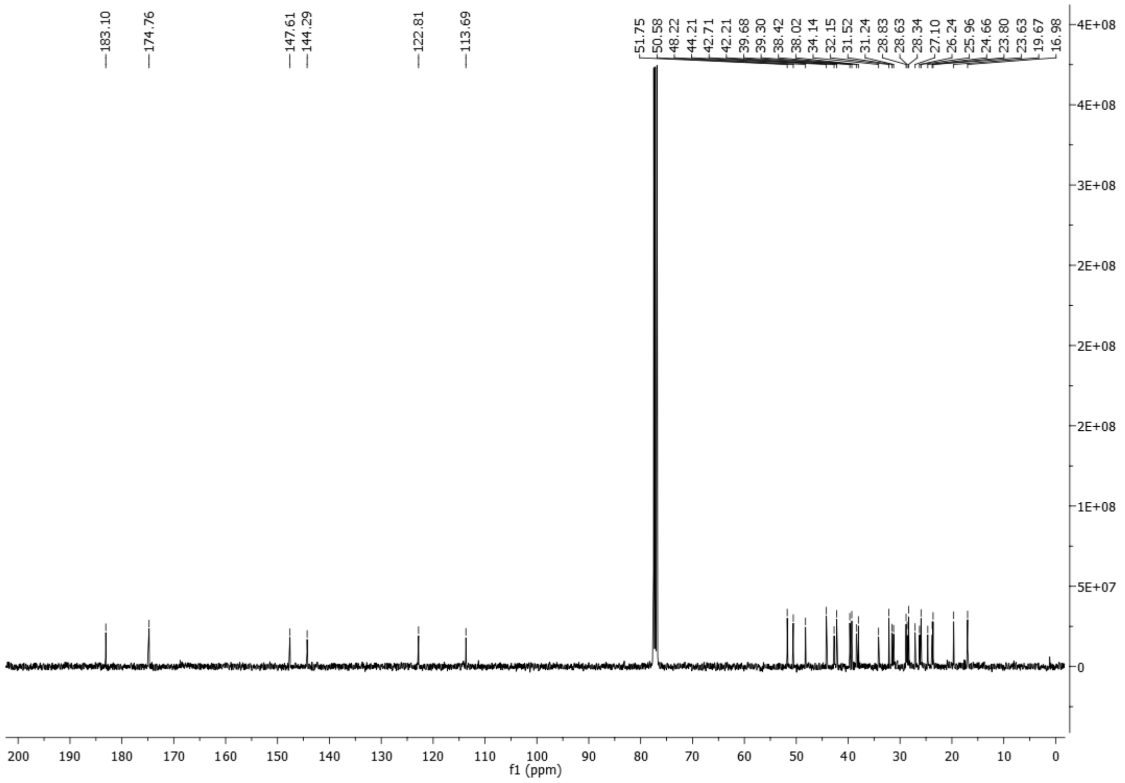

5.3. DEPT-135 spectrum for compound 11 recorded in $CDCl_3$

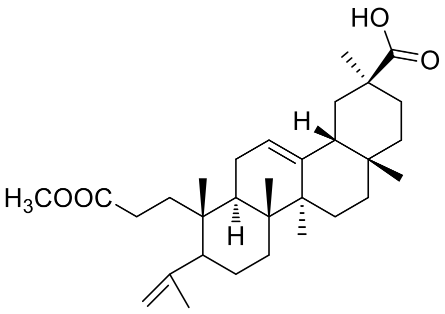

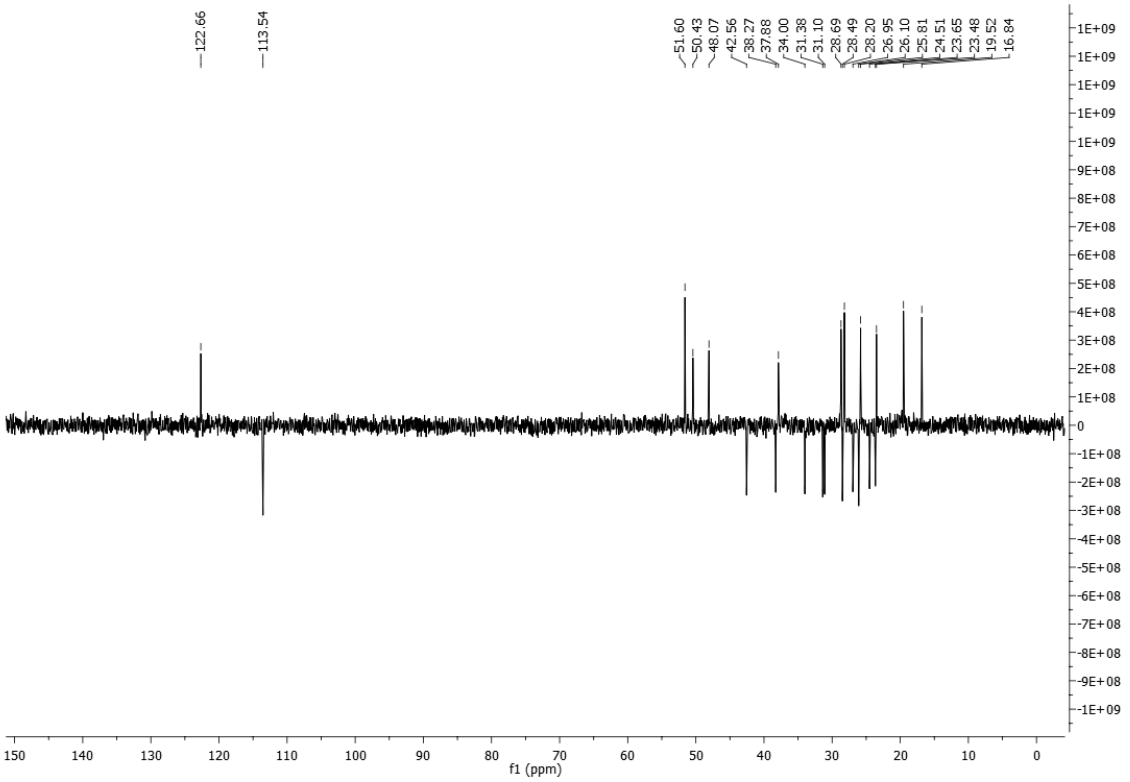

6. NMR data for compound 12

6.1. ^{1}H NMR spectrum for compound 12 recorded in $CDCl_3$

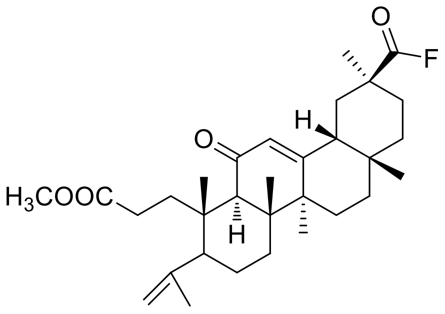

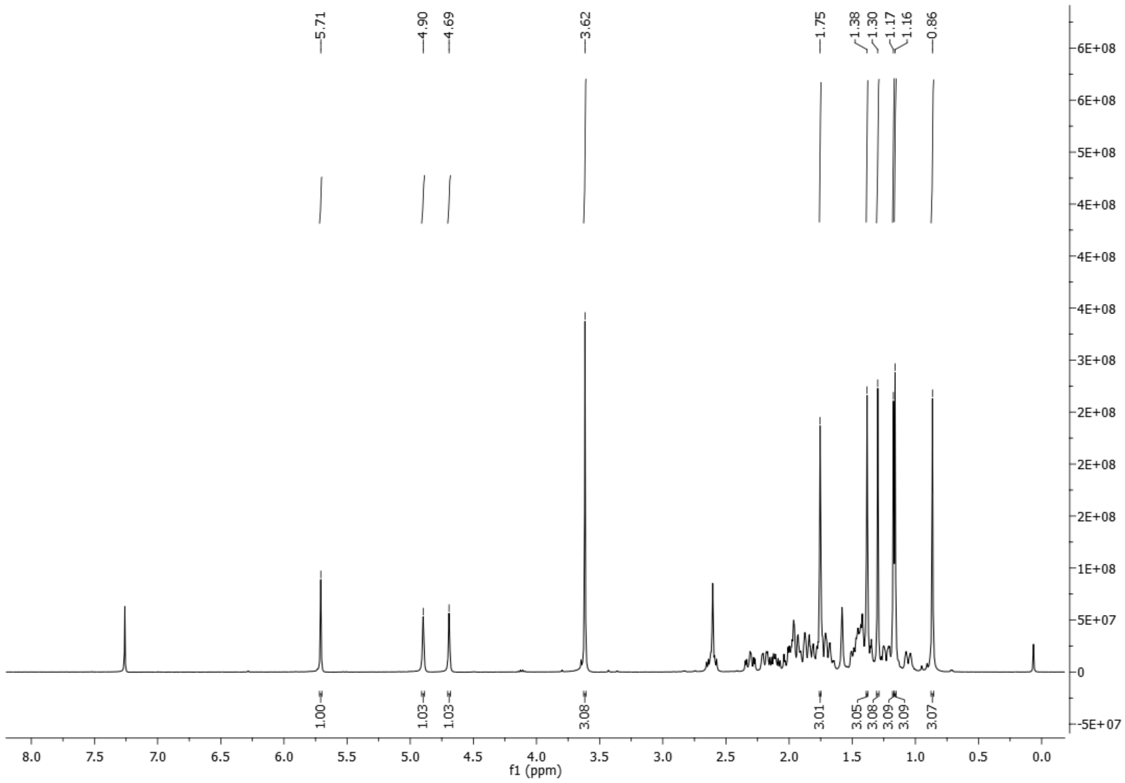

6.2. ^{13}C NMR spectrum for compound 12 recorded in $CDCl_3$

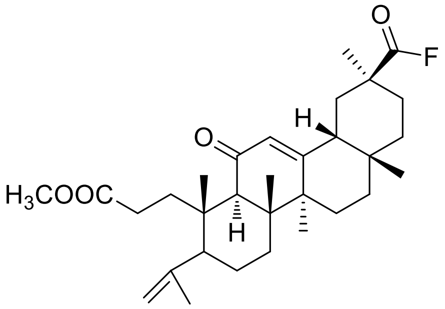

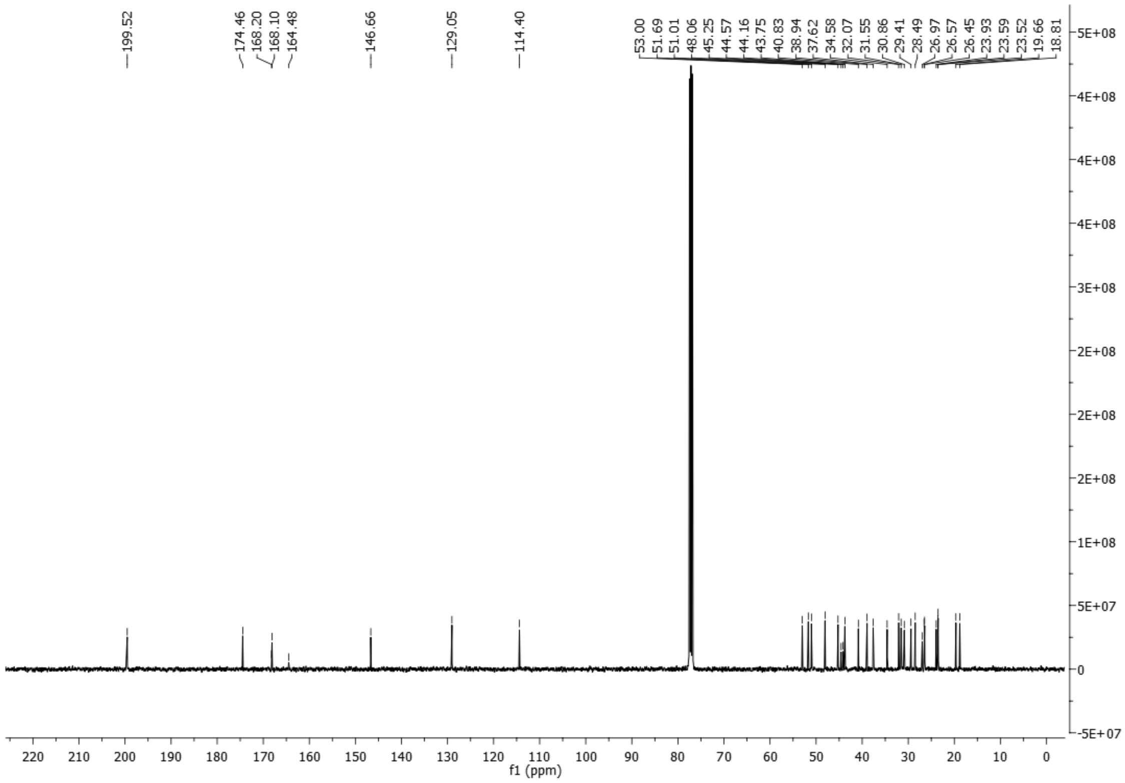

6.3. DEPT-135 spectrum for compound 12 recorded in $CDCl_3$

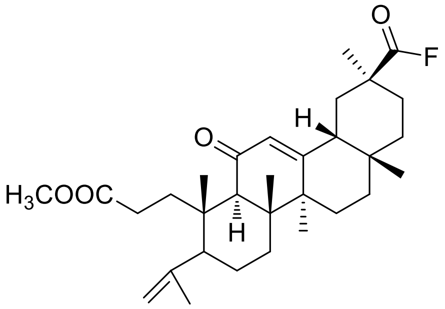

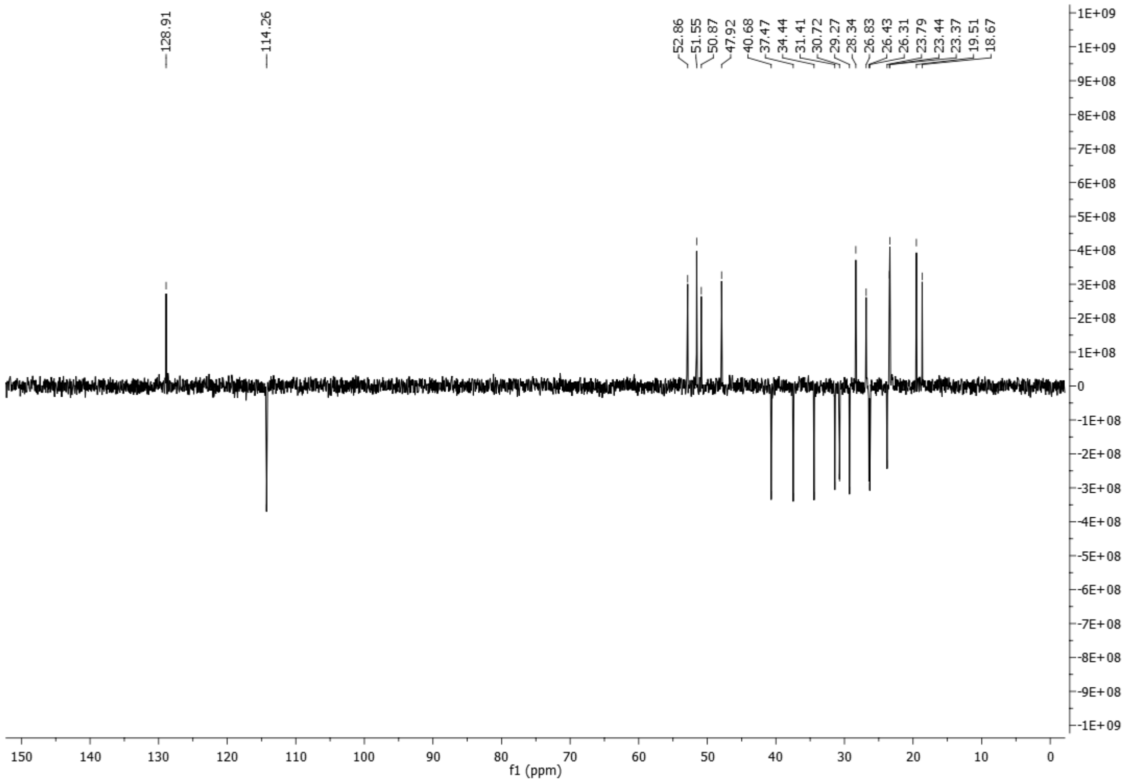

7. NMR data for compound 14

7.1. ^{1}H NMR spectrum for compound 14 recorded in $CDCl_3$

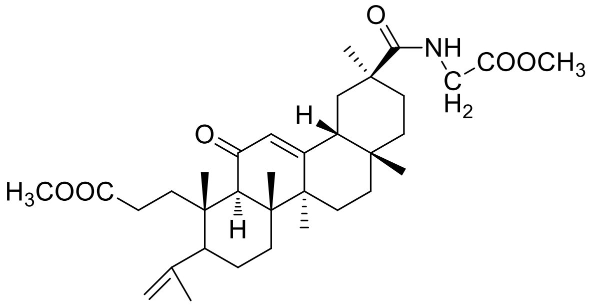

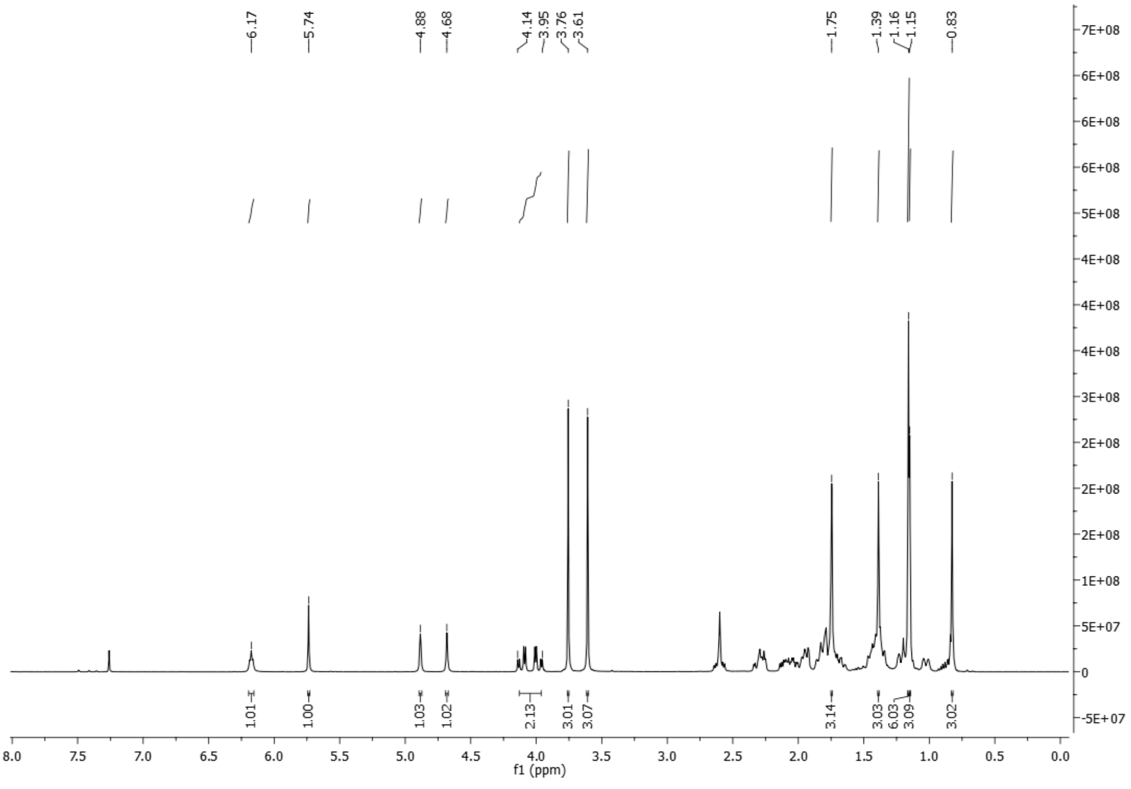

7.2. ^{13}C NMR spectrum for compound 14 recorded in $CDCl_3$

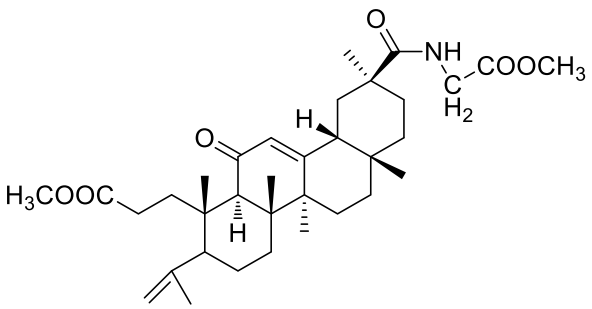

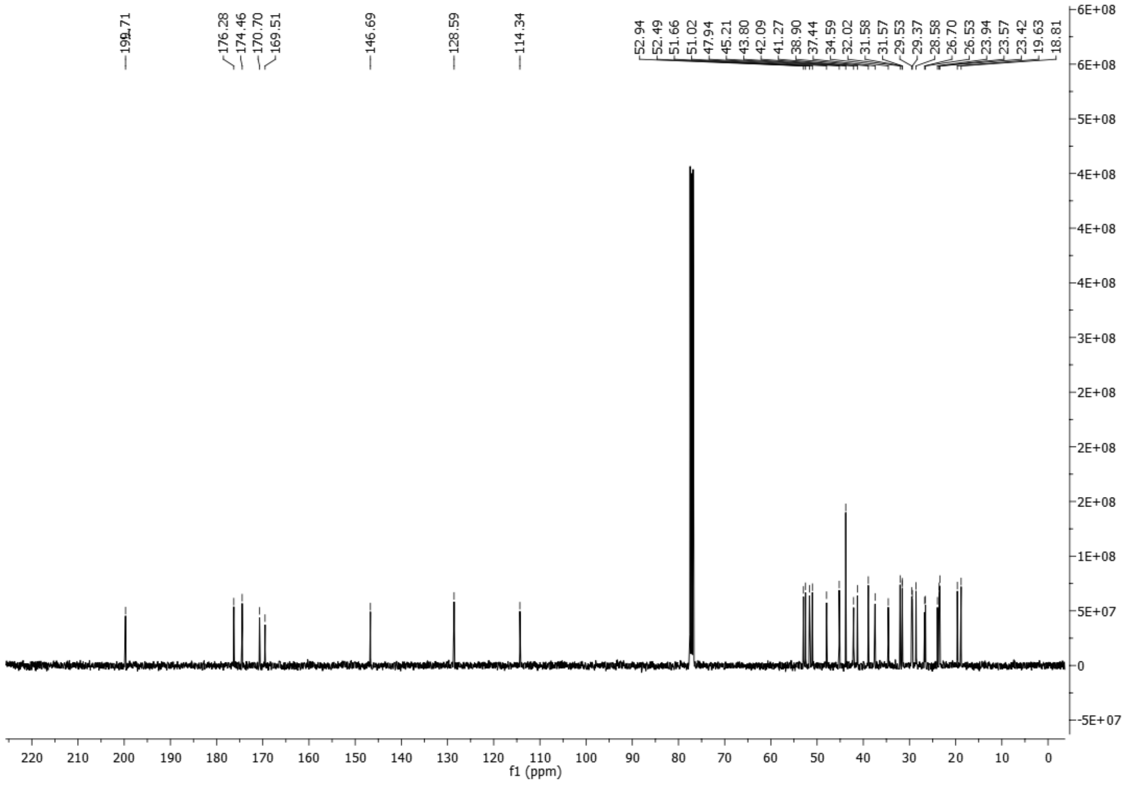

7.3. DEPT-135 spectrum for compound 14 recorded in $CDCl_3$

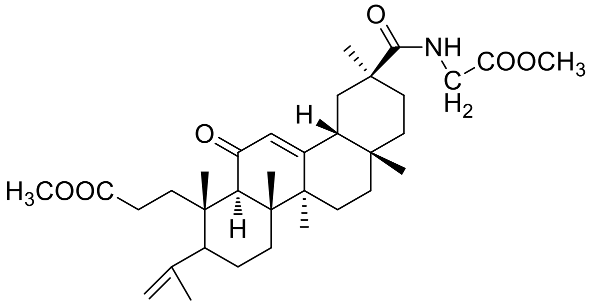

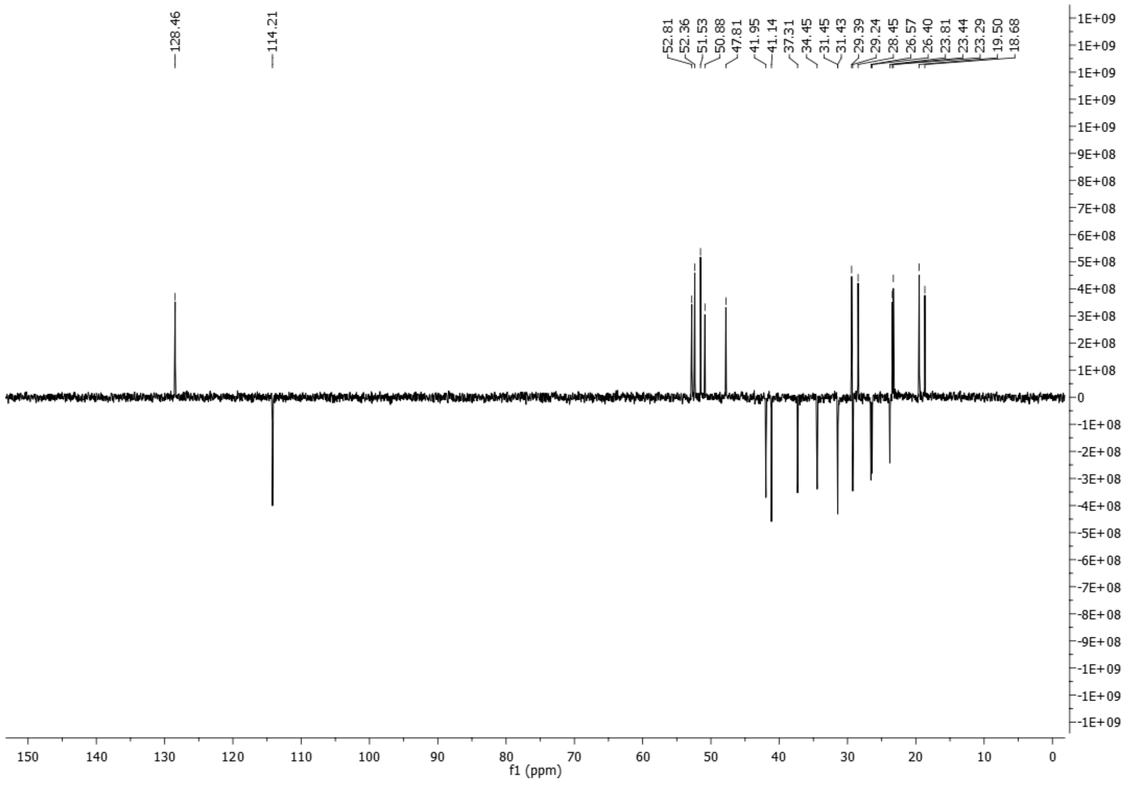

8. NMR data for compound 17

8.1. ^{1}H NMR spectrum for compound 17 recorded in $CDCl_3$

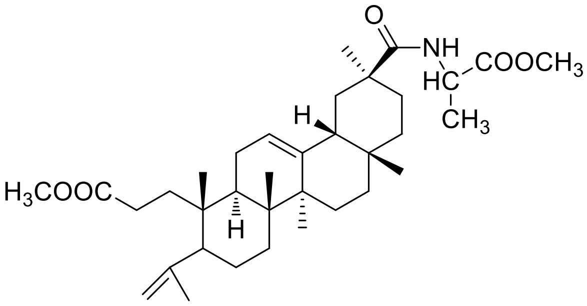

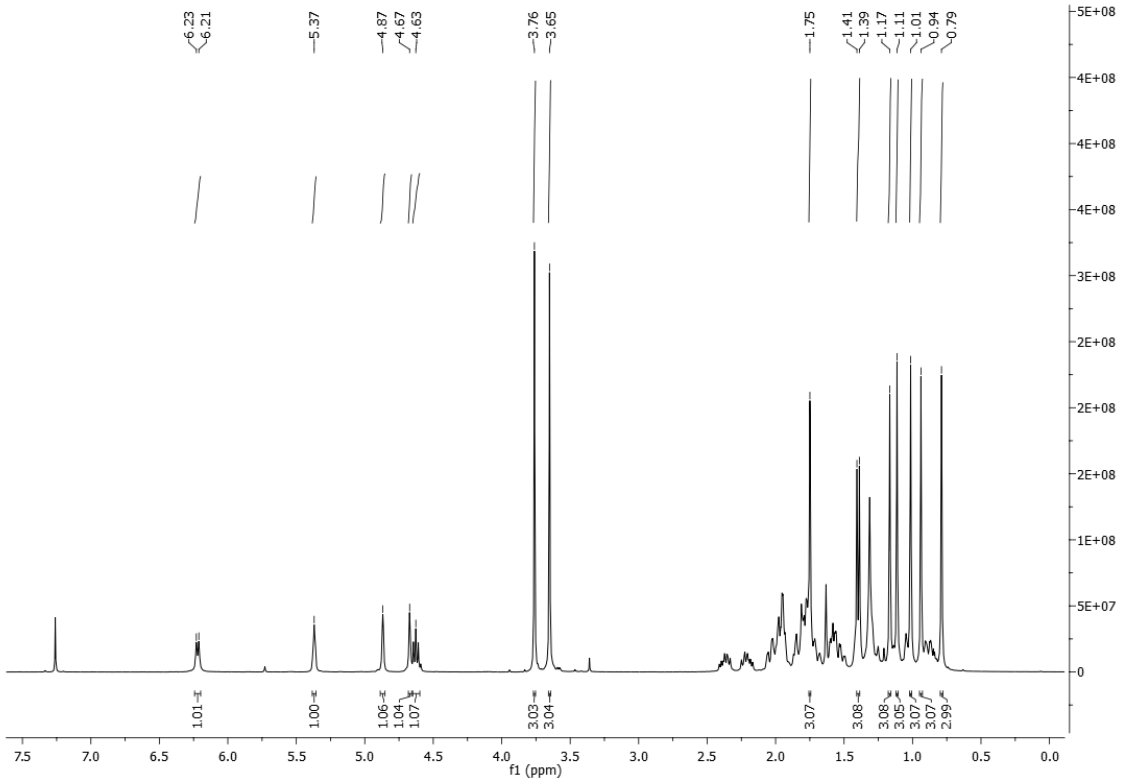

8.2. ^{13}C NMR spectrum for compound 17 recorded in $CDCl_3$

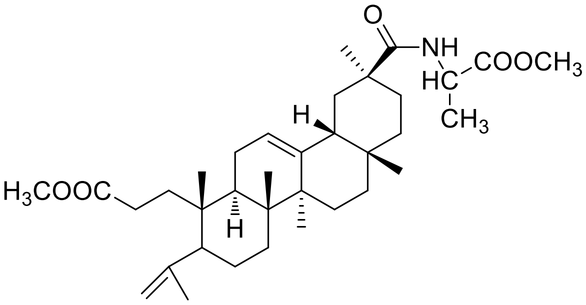

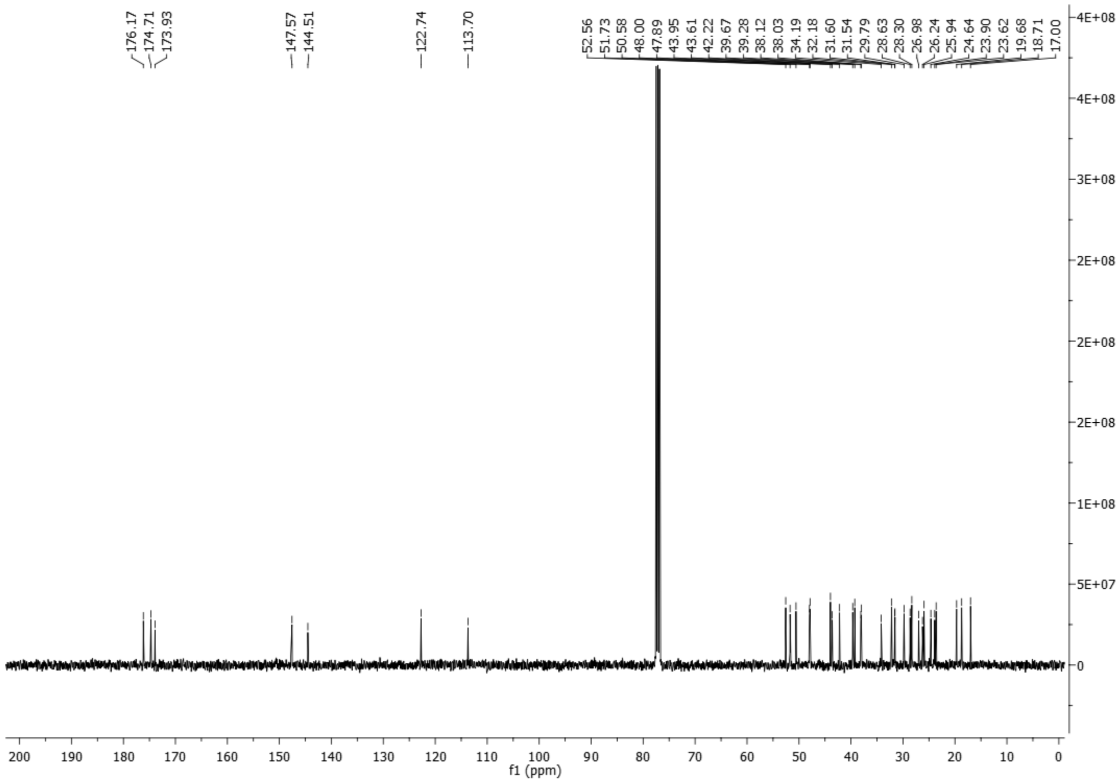

8.3. DEPT-135 spectrum for compound 17 recorded in $CDCl_3$

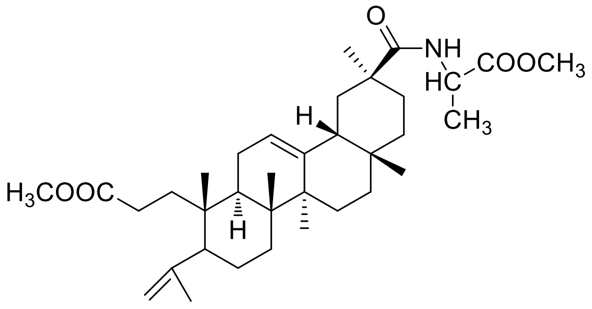

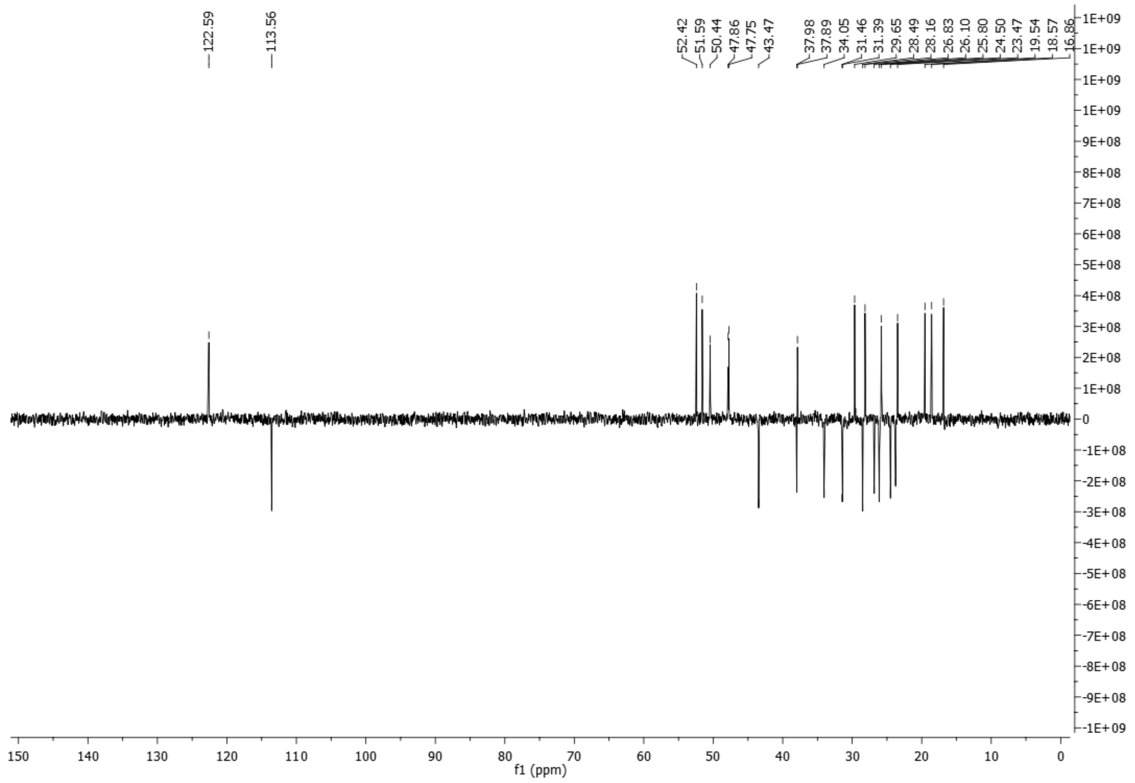

9. NMR data for compound 18

9.1. ^{1}H NMR spectrum for compound 18 recorded in $CDCl_3$

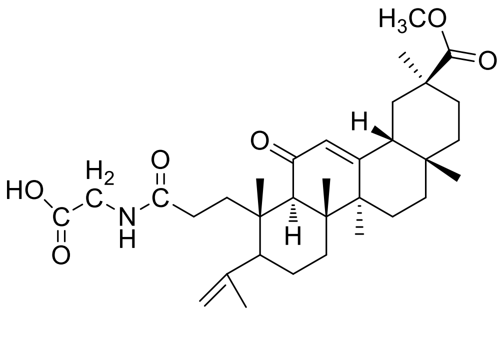

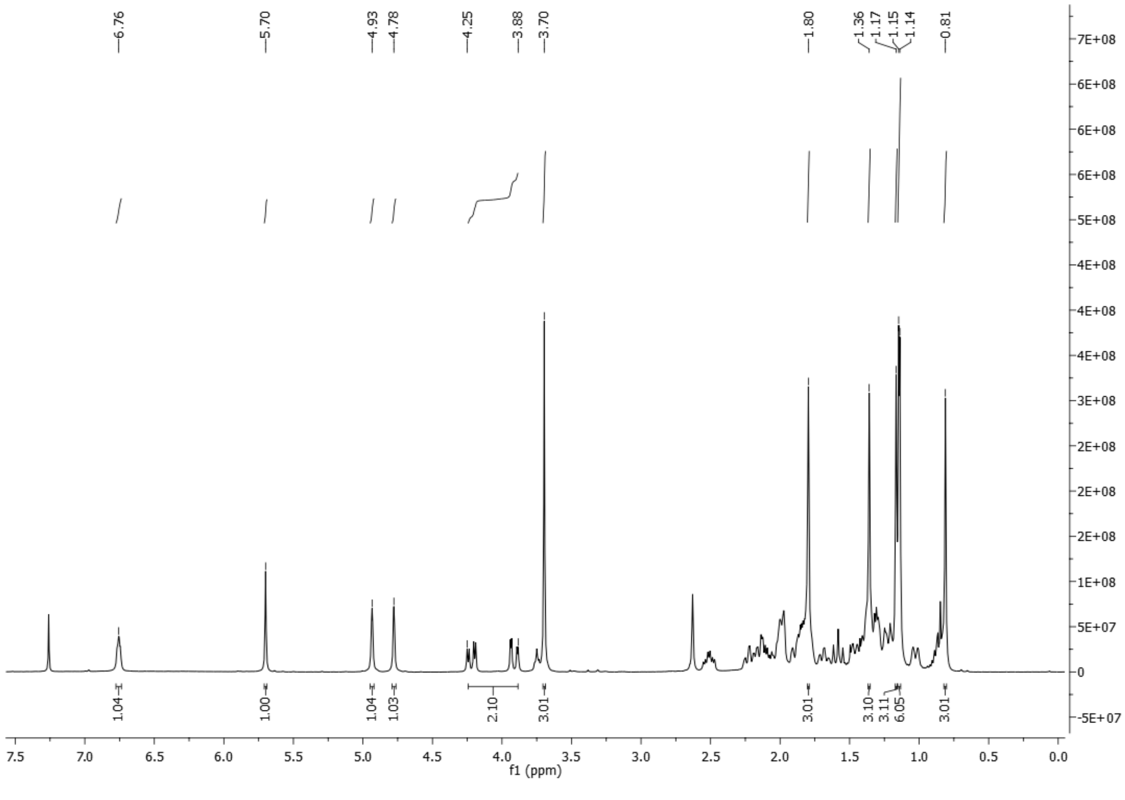

9.2. ^{13}C NMR spectrum for compound 18 recorded in $CDCl_3$

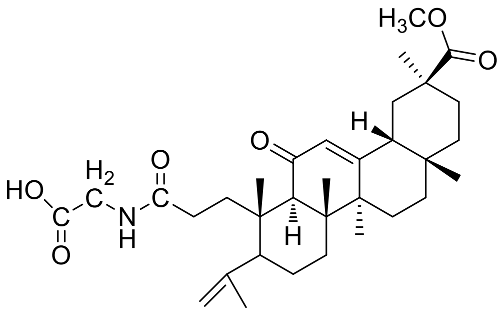

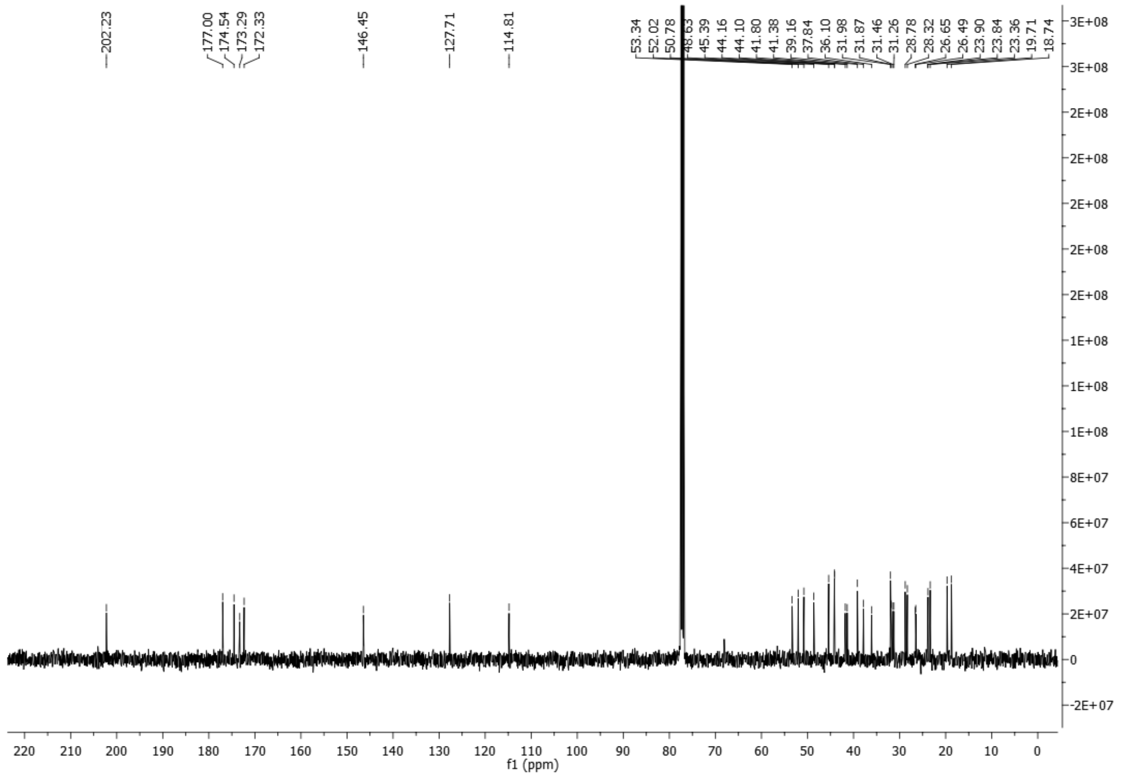

9.3. DEPT-135 spectrum for compound 18 recorded in $CDCl_3$

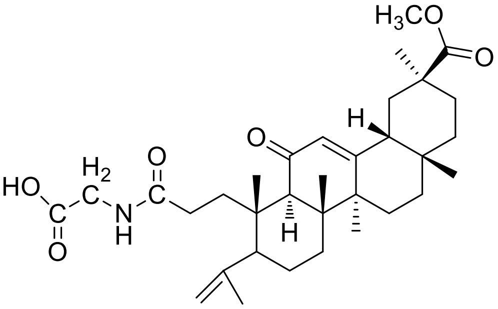

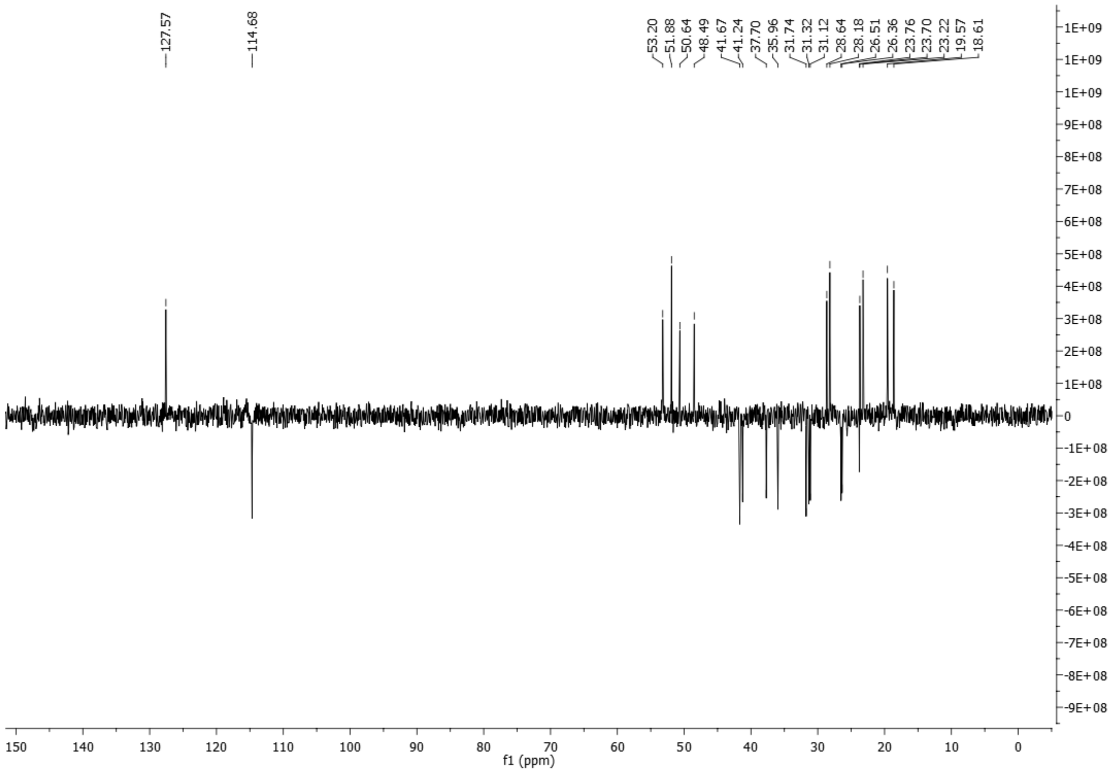

10. NMR data for compound 19

10.1. ^{1}H NMR spectrum for compound 19 recorded in $CDCl_3$

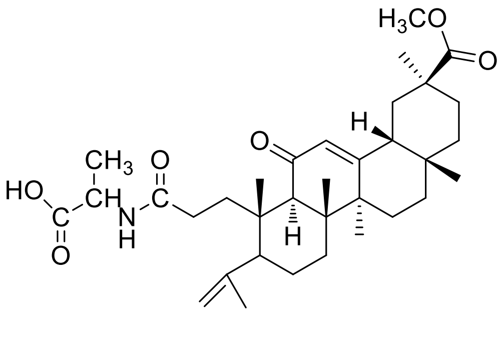

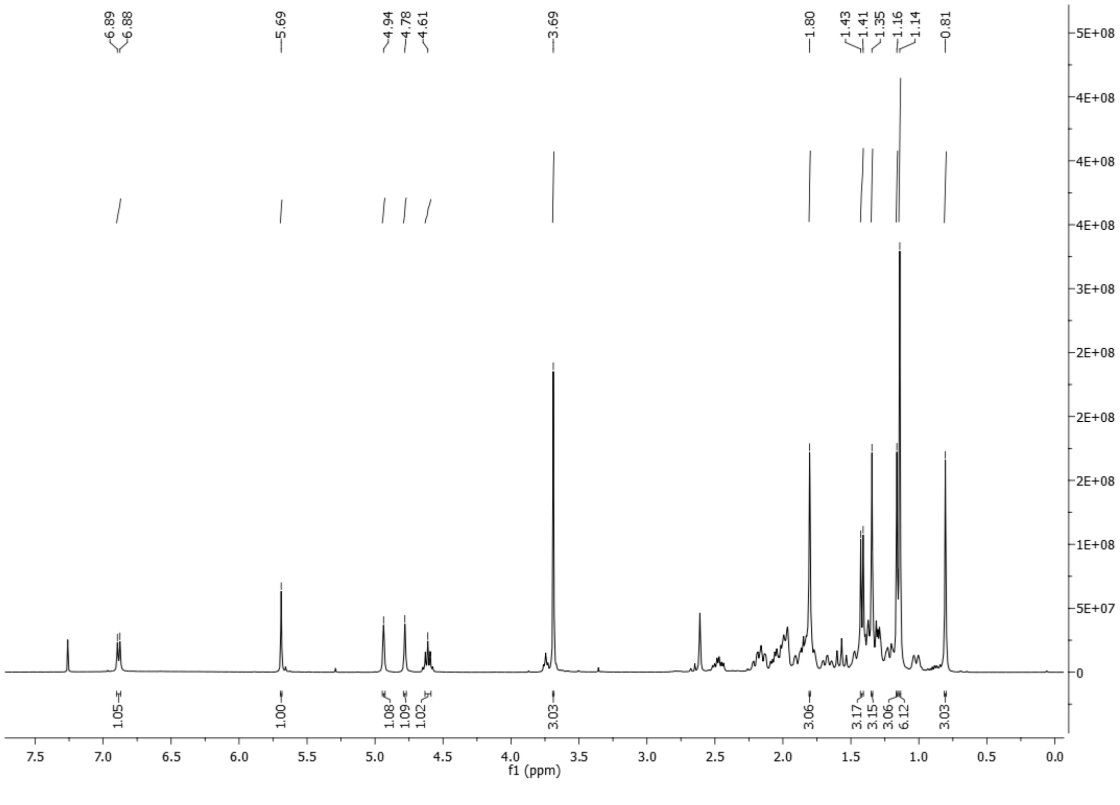

10.2. ^{13}C NMR spectrum for compound 19 recorded in $CDCl_3$

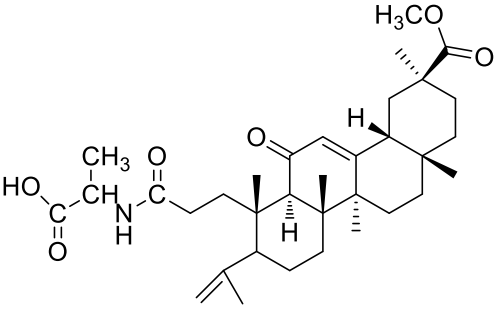

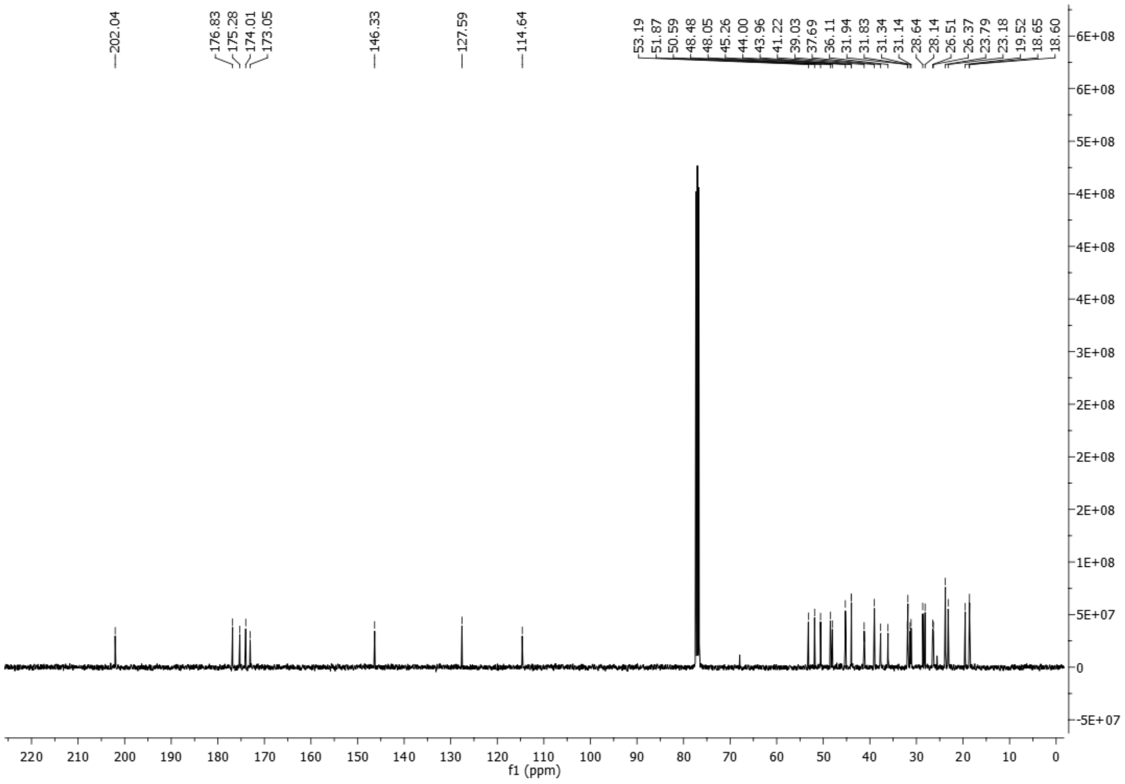

10.3. DEPT-135 spectrum for compound 19 recorded in $CDCl_3$

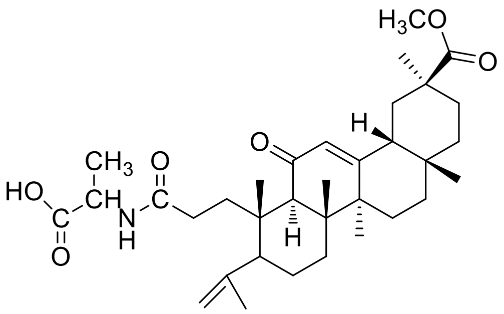

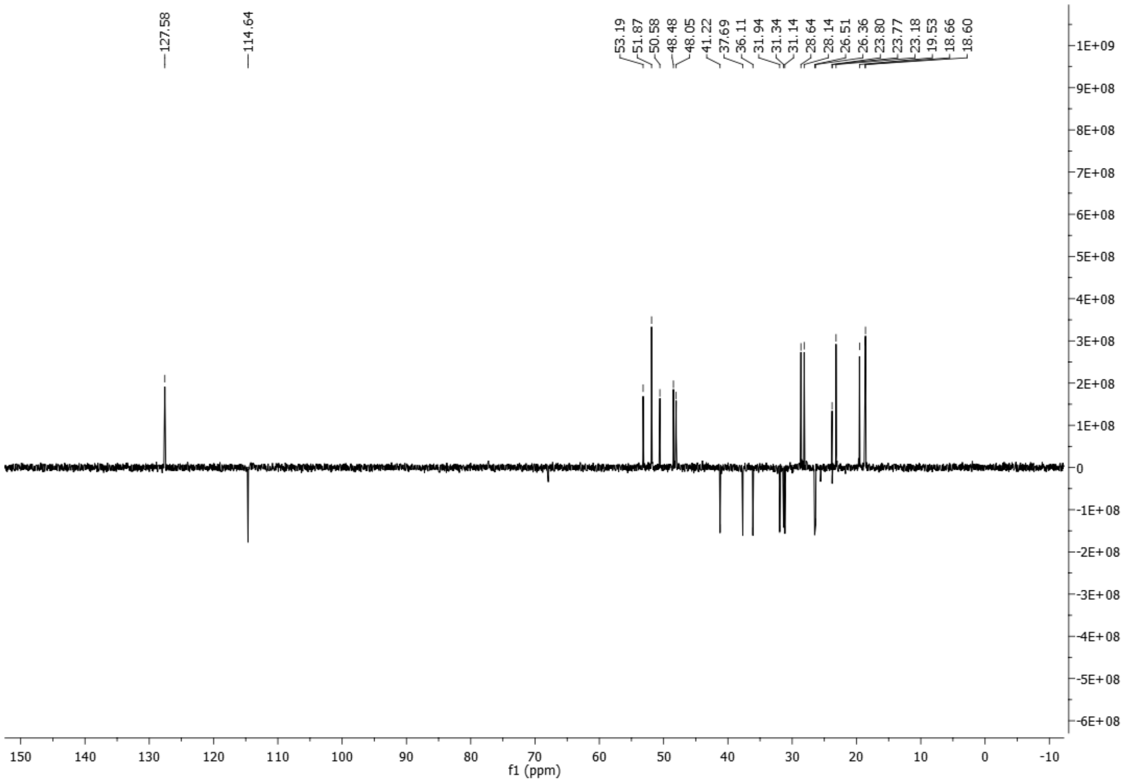

11. NMR data for compound 20

11.1. ^{1}H NMR spectrum for compound 20 recorded in $CDCl_3$

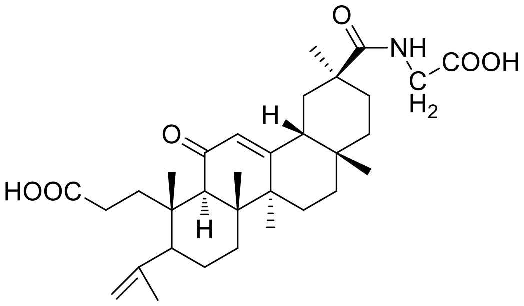

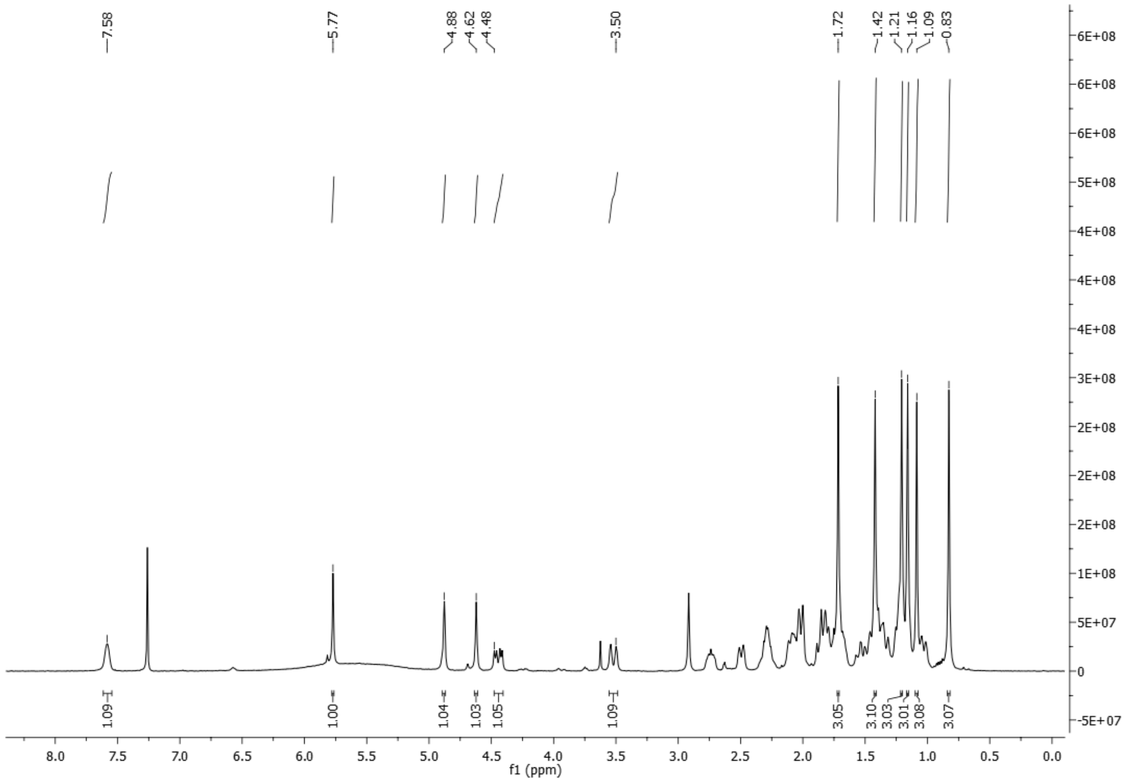

11.2. ^{13}C NMR spectrum for compound 20 recorded in $CDCl_3$

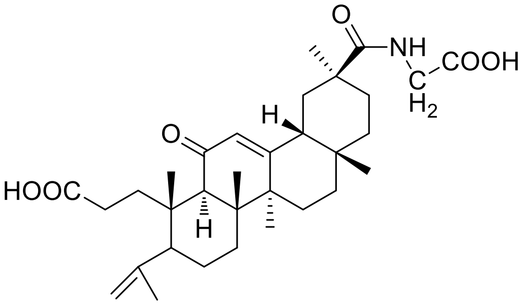

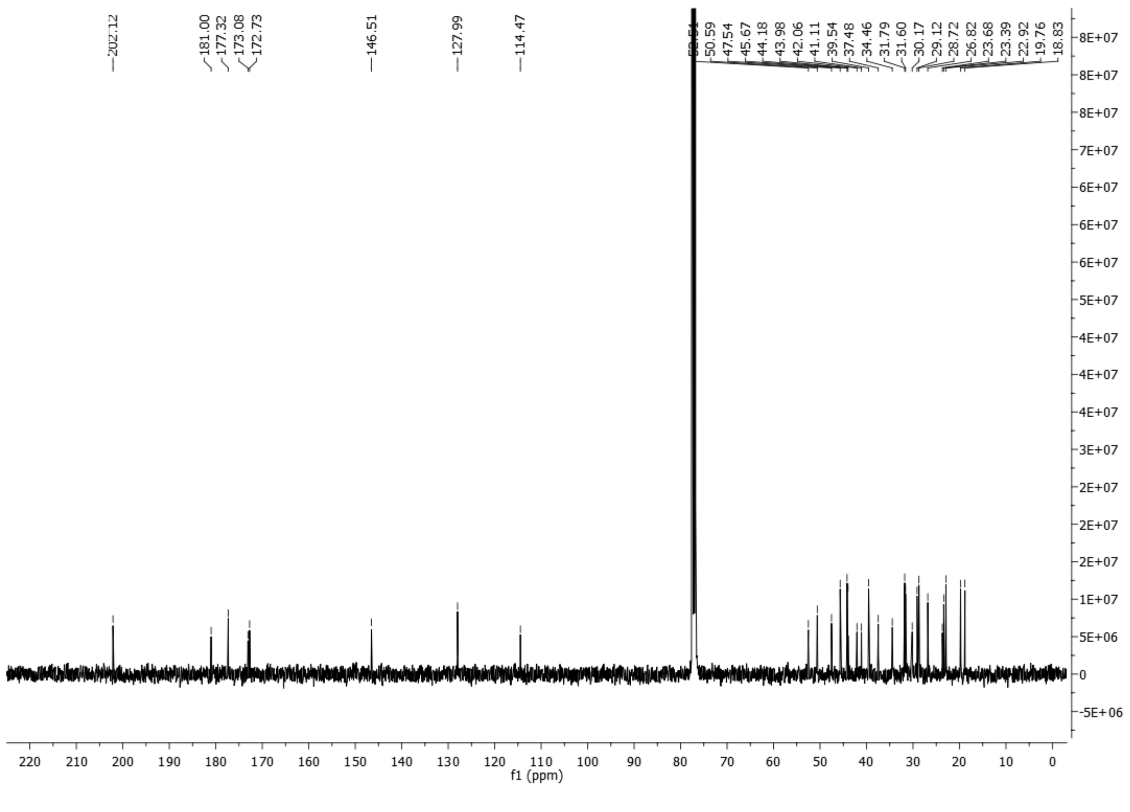

11.3. DEPT-135 spectrum for compound 20 recorded in $CDCl_3$

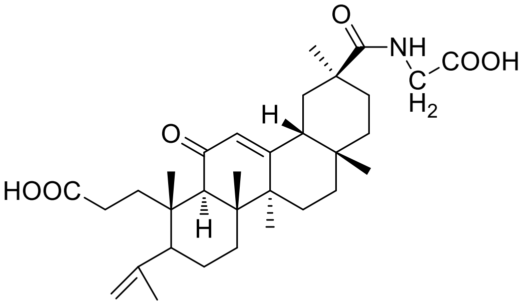

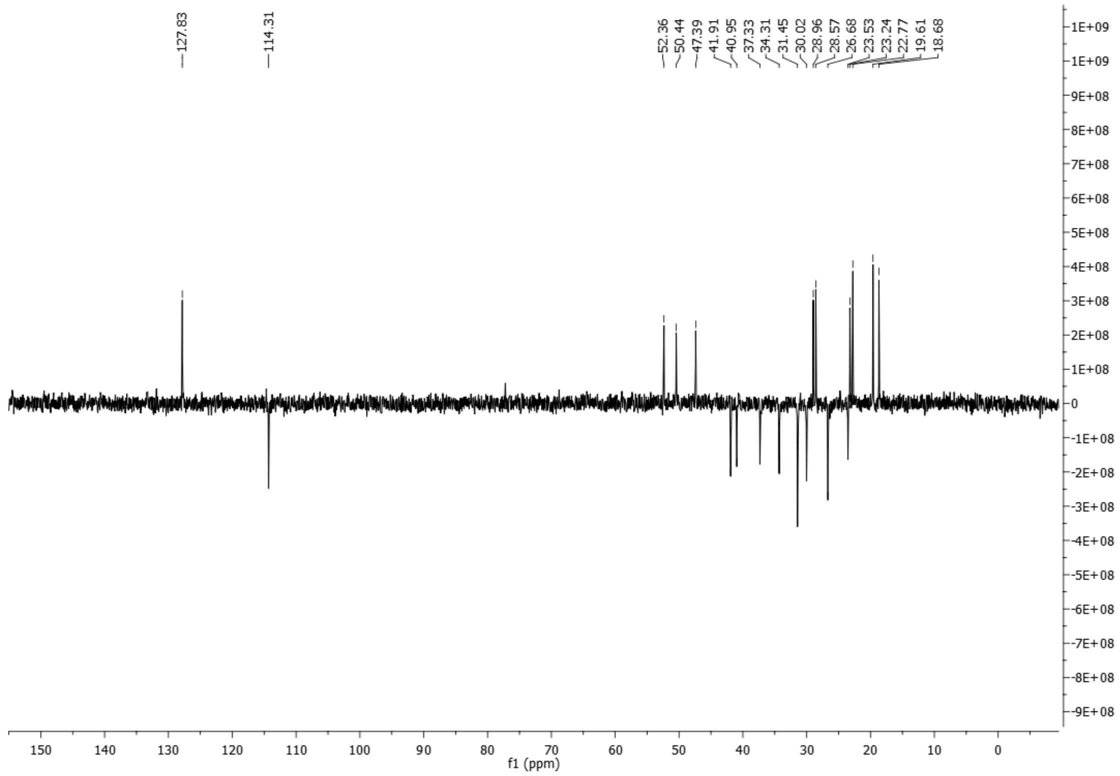

Supplement: Supplementary file 1 [file molecules-24-02938-s001.pdf]
